# Comparative techno-economic optimization of microgrid configurations using hybrid battery–hydrogen storage: NEOM case study, Saudi Arabia

**DOI:** 10.1371/journal.pone.0326050

**Published:** 2025-09-05

**Authors:** Abdullah M. Alharbi, Ziad M. Ali, Ahmed A. Zaki Diab

**Affiliations:** 1 Department of Electrical Engineering, College of Engineering in Wadi Alddawasir, Prince Sattam Bin Abdulaziz University, Wadi Alddawasir, Saudi Arabia; 2 Electrical Engineering Determent, Faculty of Engineering, Minia University, Minia, Egypt; Yalova University, TÜRKIYE

## Abstract

Renewable energy systems are at the core of global efforts to reduce greenhouse gas (GHG) emissions and to combat climate change. Focusing on the role of energy storage in enhancing dependability and efficiency, this paper investigates the design and optimization of a completely sustainable hybrid energy system. Furthermore, hybrid storage systems have been used to evaluate their viability and cost-benefits. Examined under a 100% renewable energy microgrid framework, three setup configurations are as follows: (1) photovoltaic (PV) and Battery Storage System (BSS), (2) Hybrid PV/Wind Turbine (WT)/BSS, and (3) Integrated PV/WT/BSS/Electrolyzer/Hydrogen Tank/Fuel Cell (FC). Using its geographical solar irradiance and wind speed data, this paper inspires on an industrial community in Neom, Saudi Arabia. HOMER software evaluates technical and economic aspects, net present cost (NPC), levelized cost of energy (COE), and operating costs. The results indicate that the PV/BSS configuration offers the most sustainable solution, with a net present cost (NPC) of $2.42M and a levelized cost of electricity (LCOE) of $0.112/kWh, achieving zero emissions. However, it has lower reliability, as validated by the provided LPSP. In contrast, the PV/WT/BSS/Elec/FC system, with a higher NPC of $2.30M and LCOE of $0.106/kWh, provides improved energy dependability. The PV/WT/BSS system, with an NPC of $2.11M and LCOE of $0.0968/kWh, offers a slightly lower cost but does not provide the same level of reliability. The surplus energy has been implemented for hydrogen production. A sensitivity analysis was performed to evaluate the impact of uncertainties in renewable resource availability and economic parameters. The results demonstrate significant variability in system performance across different scenarios.

## 1. Introduction

### 1.1 Research motivation

**Fig 1:**
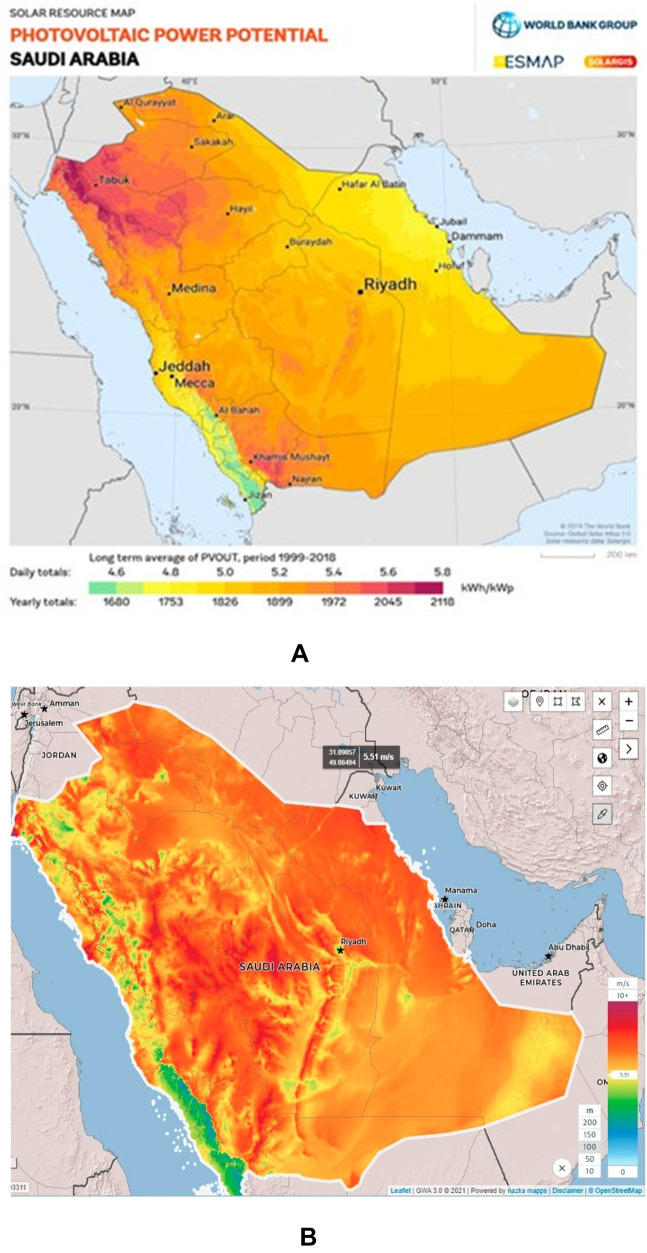
a) Map illustrating the solar photovoltaic potential of Saudi Arabia; Map obtained from the “Global Solar Atlas 2.0, a free, web-based application is developed and operated by the company Solargis s.r.o. on behalf of the World Bank Group, utilizing Solargis data, with funding provided by the Energy Sector Management Assistance Program (ESMAP). For additional information: https://globalsolaratlas.info, and b) Map illustrating wind speed and wind production potential in Saudi Arabia [3]; Map obtained from the Global Wind Atlas version 4.0, a free, web-based application developed, owned and operated by the Technical University of Denmark (DTU). The Global Wind Atlas version 4.0 is released in partnership with the World Bank Group, utilizing data provided by Vortex, using funding provided by the Energy Sector Management Assistance Program (ESMAP). For additional information: https://globalwindatlas.info”.

The Kingdom of Saudi Arabia has developed a strong strategy to enhance energy dependence on renewable energy resources to address climate change. This initiative seeks to diminish dependence on fossil fuels and alleviate thermal emissions. The Kingdom is making notable progress by investing in solar and wind projects to meet its renewable energy objectives and reduce its reliance on non-renewable sources. Saudi Arabia has set a strategic objective to produce 50% of its electricity from renewable sources by 2030. In February 2019, the kingdom revised its renewable energy targets, aiming for 27.3 GW of capacity within five years and 58.7 GW by 2030. The BP Statistical Review of World Energy 2022 reported that renewables contributed only 0.2% to the nation’s electricity generation of 356.6 TWh. Solar energy accounted for 0.8 TWh, while natural gas and oil dominated the energy mix with 215.9 TWh and 139.9 TWh, respectively [1]. KSA has a plan to implement a 21 GW of renewable energy projects, primarily focusing on solar energy. These projects ensure the Saudi Arabia’s goal of integrating solar and wind energy into its energy portfolio to achieve 50% renewable electricity generation by 2030 [2]. Creating entirely clean energy on a large scale for NEOM and ultimately for the entire globe. Utilizing unparalleled wind and solar resources, along with the largest hydrogen facility globally, we will foster and expedite renewable solutions. An objective is to progress beyond zero carbon towards a circular economy. Furthermore, the ruler area of RES should be meticulously designed to comprehensively cover the energy systems of all regions and communities. Fig 1.a illustrates the solar power potential of Saudi Arabia [3]. The wind speed in most parts of Saudi Arabia is highly conducive to power generation. Fig 1.b illustrates the wind speed distribution in Saudi Arabia [3].

Hybrid renewable energy systems (HRES) are crucial for the electrification of rural regions [4]. The HRES has been examined and recommended in numerous studies. However, there are needs to more analysis considering different configurations of HRES which may aids to improve the HRES reliability and minimize their costs [5]. Hybrid systems are explored to develop energy storage solutions that can effectively manage supply-demand balance and mitigate the variability of photovoltaic and wind power generation [6]. Moreover, the energy storage systems have a huge role to enhance the economic and technical feasibility of the HRES [7,8]. Furthermore, hydrogen possesses the highest specific energy among all fuels [9]. Although it is not a primary energy source, it functions as a versatile energy carrier with applications in transportation, heating, electricity generation, and various industrial processes [10]. Moreover, it can be stored for extended durations, even across seasons [11,12], making it a practical solution for remote areas lacking grid access, such as islands [12].

This paper evaluates the renewable energy potential of NEOM region in Saudi Arabia, analyzing solar and wind resources. It presents a comprehensive analysis and comparison between PV/battery, PV/WT/Battery, and PV/battery/FC-based HRES configurations, excluding diesel to focus on fully renewable solutions. Furthermore, green hydrogen is produced on-site via electrolysis from the surplus electrical energy.

### 1.2 Background and related work

A great transaction of research has been done to determine the most efficient renewable energy sources for alternative power generation systems, taking into account factors such as the availability of resources and energy demands specific to locations [13,14]. Due to their renewable nature and minimal environmental impact, photovoltaic (PV) systems have been widely adopted globally [15,16]. PV generation is influenced by solar irradiance, temperature, and daylight hours, which means that energy storage is needed to make up for less sunlight at night, leading to the need for larger battery capacities [17,18]. Diesel generators are a popular backup power source for critical loads in remote areas where fuel transportation is difficult, but they are costly and can present logistical challenges [19,20].

To address these challenges, HRES that combine multiple energy sources, such as photovoltaic (PV), wind, biomass, hydropower, and fuel cells, have been commonly investigated [21,22]. However, both PV and wind energy are characteristically unpredictable, as their output power is influenced with the weather conditions [23,24]. To mitigate this variability, the integration of proton exchange membrane fuel cells (PEMFCs) with PV or wind systems can significantly improve system stability and reliability [25]. PEMFCs generate electricity through an electrochemical process, producing power without greenhouse gas emissions when hydrogen is used as the fuel [26]. Despite this limitation, PEMFCs has advantages, including quiet operation, low maintenance requirements, and high efficiency, making them a appreciated component in HRESs [27].

Hydrogen combustion generators are sometimes considered as an alternative, but they have some major downsides—they’re noisy, produce more emissions, and need frequent maintenance. In contrast, fuel-cell-based hybrid renewable systems have gotten a lot of interest from researchers lately. Studies have looked at PV-battery-fuel-cell combos, both off-grid and grid-tied [28]. Moreover, ahybrid system with fuel cells, solar panels (PV), and batteries was rolled out in remote parts of France—and it actually boosted performance while cutting costs [29]. Another project developed a smarter energy management system for a grid-linked setup using PEM fuel cells, PV panels, batteries, and supercapacitors [30]. Researchers have experimented with dynamic optimization methods—particle swarm optimization (PSO), for example—to tackle three key issues in hybrid systems: cost (NPC), energy gaps, and emissions. These systems combine everything from wind turbines and solar panels to diesel backups, batteries, fuel cells, electrolyzers, and even hydrogen storage [31]. Additional research has focused on evaluating the initial costs, NPC, and levelized energy costs for PV-fuel cell-battery systems designed to power remote communities, demonstrating the viability of HRES as a reliable and sustainable energy solution [32].

A HRES has been optimally introduced to meet the peak load demands of 10–100 kW considering zero greenhouse gas emissions [33]. The system integrates photovoltaic (PV) modules, wind turbines, battery storage, and a hydrogen production and storage unit. The results shows that a solar-wind hybrid system coupled with battery storage is most efficient for meeting short-term energy needs. However, hydrogen storage developed as a more cost-effective solution during periods of insufficient solar and wind resource [34]. This study validated the ability of hybrid battery and hydrogen storage systems can enhance the reliability of the HRES under varying resource conditions.

In a separate study, HOMER software had been used for optimizing a standalone hybrid system integrating PV and fuel cells (FC), excluding battery storage, to meet the demand of a city [35]. The results show that fuel cells aid as a feasible, low-maintenance, and environmentally sustainable substitute the diesel generators [34,36,37]. Similarly, a hybrid PV/wind/FC system has been simulated for remote locations, integrating both an electrolyzer and a reformer [38]. The simulation results confirmed that the presented hybrid configuration is a feasible reliable power source to off-grid areas.

A comparative analysis was conducted between two system configurations of the FC/PV/battery and PV/battery systems for rural electrification, with both systems evaluated against conventional diesel-based systems. Through optimization and sensitivity analysis, it was determined that PV/battery systems offer greater economic efficiency and environmental sustainability compared to diesel-based systems. These results prove that the PV/battery systems are suitable source for remote areas [21,35,39].

A feasibility study on large-scale load demand identified wind energy as cost-effective renewable energy source, and better than the photovoltaic (PV) systems in economic viability. Autonomous hybrid systems integrating wind, PV, and battery storage were optimized using an eco-design approach, focusing on achieving environmental sustainability and economic efficiency. This novel presented a methodology for probabilistic power flow analysis in hybrid wind-PV systems was also introduced. Various optimization algorithms have been applied to optimize the HRES considering feasibility analysis as introduced in [18,22,40–44].

The economic viability of renewable energy systems incorporating photovoltaic (PV) panels, electrolyzers, fuel cells (FC), and hydrogen storage tanks, specifically designed for single residential units in North America has been explored and compared [36]. Six systems of PV/battery, wind/battery, PV/wind/battery, PV/FC, wind/FC, and PV/wind/FC—in several locations in Saudi Arabia to find the configuration with the lowest COE have been examined. Their findings showed that the PV/wind/FC combination had the minimum levelized COE [45]. In another study, five hybrid PV-wind-diesel systems using hydrogen as a fuel analyzed for the diesel generator to meet a home’s electricity needs, identifying the wind/hydrogen/battery setup as the most cost-effective configuration [46]. An electrolyzer, hydrogen tanks, and FC could serve as a safe, compact, and reliable backup power system for a house has been demonstrated in Mexico [47]. A solar-hydrogen reversible fuel cell for residential use, noting that significant cost reductions are necessary to compete with conventional energy storage options experimentally evaluated [48].

The technical, economic, and environmental performance of various hybrid power systems for remote telecommunication stations have been evaluated in [49]. Using HOMER Software, different configurations were analyzed to identify the most cost-effective setup based on net present cost (NPC) and cost of energy (COE) [49]. Jansen et al. conducted a case study on powering telecommunication base stations near Dakar, Senegal, with an autonomous renewable energy microgrid. The system combined solar photovoltaic (PV) and wind turbine (WT) technologies for electricity generation, supported by a regenerative hydrogen fuel cell providing backup power for up to 10 days [50]. A study in [50] examined the feasibility and environmental impact of hybrid systems integrating PV, wind, and diesel generator (DG) technologies. The research focused on powering telecommunication base transceiver stations in rural areas of the Democratic Republic of Congo. Zegueur et al. performed a techno-economic analysis of a hybrid PV-WT-DG system designed to supply power to a rural telecommunication station in northeastern Algeria [51]. Another study developed an optimization algorithm for a hydrogen-based energy storage system, aiming to reduce renewable energy source (RES) curtailment. Applied to an independent power network in the Aegean Sea, the system effectively utilized surplus RES energy for grid support and transportation, achieving up to 39% utilization of otherwise wasted renewable power [52].

Table 1 shows an examination of research endeavors to validate the diversity of employed methodologies and platforms, together with the range of proposed microgrid designs. Researchers in [53–57] illustrate the techno-economic and environmental viability of hybrid renewable energy systems for hydrogen production and storage. Reference [1] delineates the efficacy of hybrid PV/Wind systems across various locales, with Mersa Matruh yielding 118,115 kWh/year of electricity and 1,972 kg/year of hydrogen, Aswan generating 107,285 kWh/year and 1,795 kg/year of hydrogen, and Cairo producing 84,096 kWh/year and 1,418 kg/year of hydrogen. The levelized cost of hydrogen (LCOH) in this study varied between $4.54/kg and $7.48/kg, signifying competitive hydrogen production expenses. Reference [54] examines extensive hydrogen production, demonstrating that Dakhla, Morocco, attained the lowest Levelized Cost of Hydrogen (LCOH) at $2.54/kg via an optimal integration of solar panels and wind turbines. Their findings underscore the viability of integrating solar panels and wind turbines for economical hydrogen production, noting that the expense of water desalination is minimal, constituting about 0.12% to 0.35% of the net present expenditures. Reference [55] highlights the efficacy of PV/Wind/Diesel/Battery/Electrolyzer systems in co-generating electricity and hydrogen, attaining an ideal ecological footprint with energy priced at $0.252/kWh and hydrogen at $2.59/kg. Reference [56] examines the scalability of renewable energy systems, detailing a 100 MW photovoltaic system that generates 158,484–175,675 MWh annually and produces 2,524–2,761 tonnes of hydrogen per year, with a levelized cost of hydrogen (LCOH) ranging from 22.54 to 28.38 CNY/kg and an energy efficiency of 9.03–9.31%. Ref. [57] demonstrates the environmental and economic advantages of a hydrogen-methane thermal power plant, which attained a 16-year payback period through 100% hydrogen combustion and an annual CO2 reduction of 155 tons.

**Table 1:** Summary of Techno-Economic Studies on Renewable Energy and Hydrogen Systems.

Ref.	Study Focus	Technologies Evaluated	Optimization Method	Key Findings	Cost Metrics
[53]	Techno-economic analysis of hybrid PV/Wind systems for hydrogen generation and storage	PV, Wind, Electrolyzer, Hydrogen Storage	Transient mathematical modeling (MATLAB/Simulink)	Hydrogen production: Mersa Matruh (1,972 kg/year), Aswan (1,795 kg/year), Cairo (1,418 kg/year); Annual electricity generation: Mersa Matruh (118,115 kWh), Aswan (107,285 kWh), Cairo (84,096 kWh)	LCOH: $4.54–$7.48/kg
[54]	Techno-economic study of large-scale hydrogen production via hybrid PV/Wind systems	PV, Wind, Electrolyzer, Hydrogen Storage	HOMER Pro	Dakhla, Morocco, achieved the lowest LCOH of $2.54/kg using an optimized combination of solar arrays and wind farms	LCOH: $2.54/kg
[55]	Techno-economic and environmental assessment of PV/Wind/Diesel/Battery/Electrolyzer systems	PV, Wind, Diesel, Battery, Electrolyzer	Multi-criteria decision-making	PV/Wind/Diesel/Battery/Electrolyzer system identified as optimal for co-generation of electricity and hydrogen, with the lowest hydrogen production cost and reduced environmental impact	COE: $0.252/kWh; COH: $2.59/kg
[56]	Computational strategy for a 100 MW PV system coupled with hydrogen generation	PV, Electrolyzer, Hydrogen Storage	Process modeling (MATLAB)	System produced 158,484–175,675 MWh/year of electricity and 2,524–2,761 tonnes/year of hydrogen; LCOH: 22.54–28.38 CNY/kg; Energy efficiency: 9.03–9.31%	LCOH: 22.54–28.38 CNY/kg
[57]	Hydrogen-methane thermal power plant for a high school	PV, Electrolyzer, Hydrogen Storage	ON/OFF Control	System achieved a 16-year payback period with 100% hydrogen combustion; Annual CO₂ reduction: 155 tonnes	N/A
[58]	Wind energy potential for electricity and hydrogen production	Wind Turbines, Electrolyzer, Hydrogen Storage	Techno-economic analysis (No optimization)	Kousseri identified as the best location for hydrogen production; COE: $0.0578/kWh; COH: $4.3865/kg (GE 1.5SL turbine); Payback period: 2.9 years (Enercon E-48/800)	COE: $0.0578–$0.0838/kWh; COH: $4.3865–$6.5065/kg
[59]	Techno-economic and environmental assessment of hybrid PV/Wind/Diesel systems	PV, Wind, Diesel, Battery, Fuel Cell	Cuckoo Search Algorithm (CSA)	PV/Wind/Battery/Diesel system identified as most cost-effective; COE: $0.151/kWh (Idabato); CO₂ emissions reduced by 94.32% for high consumers	COE: $0.151–$0.220/kWh

### 1.3 Research gap and contribution

As introduced, while many research give an attention to optimal design of the hybrid renewable energy systems (HRES), the optimization of off-grid systems, particularly in areas with plenty of renewable resources like Neom, remains underactive. The difficulty of creating an effective off-grid storage systems with PV and Wind sources without using backup generators that can consistently satisfy energy needs while minimizing costs is addressed by this work. Moreover, To the authors’ knowledge, a few reported work existing RES100% in the literature have explored the utilization of excess energy for green hydrogen production. Consequently, this section will investigate the feasibility of employing excess energy for hydrogen fuel production. A methodology for employing the excess energy from renewable energy systems (RESs) designed to fulfill 100% of electrical demand is presented to analysis the economic viability by generating hydrogen fuel which can be utilized locally in industrial and transportation sectors.

This paper provides a comprehensive evaluation of full renewable energy system configurations including a hybrid hydrogen and battery storage systems, addressing a gap in existing research. Unlike previous studies that primarily focus on individual storage technologies, this research systematically compares two hybrid storage system to determine the most cost-effective and sustainable solution for residential applications. The contributions may include:

- Analyzing the optimal size of three HRES configurations of PV/Battery Storage System (BSS), PV/Wind Turbine (WT)/BSS, and PV/WT/BSS/Electrolyzer/Hydrogen Tank/Fuel Cell (FC) configurations, providing a comprehensive assessment of cost, efficiency, and sustainability.
- Improving the reliability of the HRES with analyzing the systems configuration with hybrid energy storage systems of battery and hydrogen storage.
- Providing 100% renewable energy systems towards sustainability and emission-free energy solutions
- Utilizing site-specific solar irradiance and wind speed data from Neom, Saudi Arabia to ensure accurate modeling and practical applicability of findings for including the regional focus with real-world data.
- A suggestion with comprehensive analysis of utilizing the excess energy into H2 production.
- Providing the optimal configuration of the HRES through optimally determining the best renewable sources to meet the load demand considering the system constraints.

### 1.4 Paper organization

This paper is organized as follows. Section 2 outlines the methodology, including the HOMER simulation tool, standalone HRES components, system configurations under study, and site-specific data. Section 3 presents the simulation results, analyzing the performance of different HRES configurations based on economic and technical criteria. Section 4 concludes the study by summarizing key findings and identifying the most cost-effective and reliable energy system for the selected region.

## 2. Methods and methodology

The integration of renewable energy sources into a multi-source hybrid system, connected to configure an off grid microgrid, presents a promising solution for optimizing electricity generation from both a technical and economic perspective. One of the primary challenges in the design of such hybrid systems is the optimization of the sizing of energy components, particularly in terms of energy costs and overall system performance [37].

The hybrid energy system evaluated in this study consists of a photovoltaic (PV) and Wind Turbine (WT) installation. Moreover, utilizing the hybrid storage systems of BSS and hydrogen has been studied and analyzed. A fuel cell (FC) is incorporated to supply electricity to the load during periods when renewable energy is unavailable. This system is specifically designed to meet the electricity demands in SA.

### 2.1 Configurations under study

**Fig 2:**
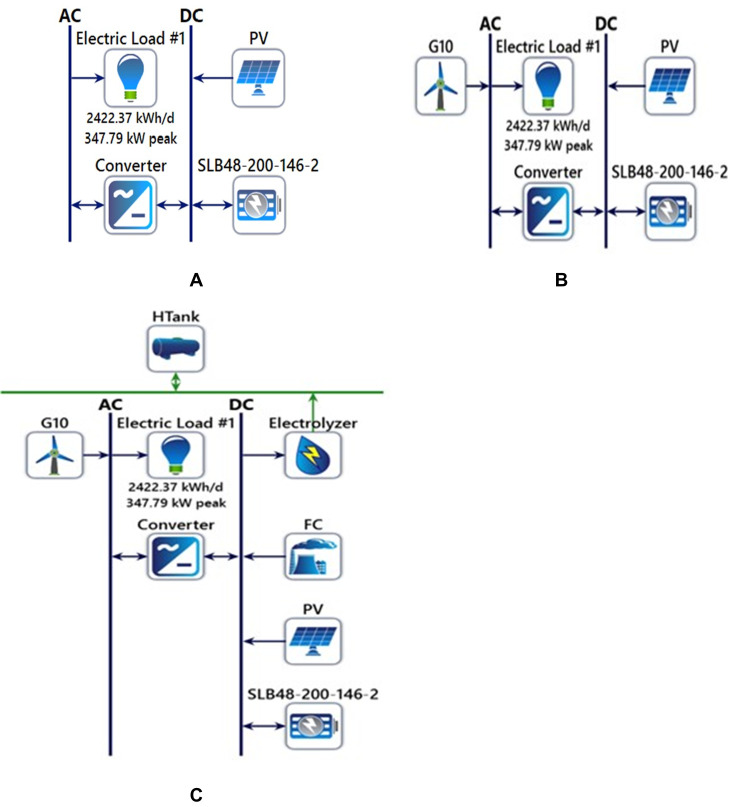
Configuration of studied MG, a) First configuration of PV/BSS, b) Second Configuration of PV/WT/BSS, and c) Third Configuration of PV/WT/BSS/Elec/FC.

Various configurations of a 100% renewable energy system have been analyzed. The first configuration consists of photovoltaic (PV) panels and battery storage, as shown in Fig 2.a. Moreover, the second configuration incorporates PV, wind turbines (WT), and battery storage as shown in Fig 2.b. The third configuration extends the system further by integrating an electrolyzer, fuel cell (FC), and hydrogen tank, in addition to the PV and battery components, as shown in Fig 2.c. All three configurations have been modeled using HOMER software.

### 2.2 Modelling of system components

#### *2.2.1* PV modeling.

The PV system converts solar energy into electricity. Its power output depends on environmental factors such as ambient temperature and solar radiation intensity [6,60]. To model and predict PV performance accurately, the following equation is used [40]:


$$P_{PV}=Y_{PV}\cdot f_{PV}\left(\frac{G_{T}}{G_{T,STC}}\right)\left[1\;+\;\alpha_{P}\;\left(T_{C}\;-\;T_{C,STC}\right)\right],$$ (1)

Where, $f_{PV}$ is the PV derating factor (%). $Y_{PV}$ is the rated capacity of the PV array (kW). $G_{T}$ represents the global solar radiation incident on the PV array at a given time step (kW/m²). $G_{T,STC}$ = 1 kW/m² is the standard incident radiation under standard test conditions (STC) at 25°C. $\alpha_{P}$ is the temperature coefficient of power (%/°C). $T_{C}$ and $T_{\text{C,STC}}$ are the PV cell temperatures under operating conditions and STC, respectively.

The PV cell temperature is calculated using:


$$T_{C}=T_{a}+\left[\frac{\left(NOCT-20\right)}{800}\right]\times G_{T},$$ (2)

where, $T_{a}$ is the ambient temperature (°C). NOCT is the nominal operating cell temperature, typically ranging from 45°C to 47°C [61].

#### 2.2.2 Wind turbine (WT) generator modeling.

The output power of a wind turbine (WT) generator depends directly on the wind speed at hub height. In practical applications, WT placement is critical to avoid obstructions such as buildings, trees, or uneven terrain, which can disrupt wind flow and reduce performance [62]. Wind speed at hub height is estimated using the power law based on measurements from an anemometer at a reference height. WT power output is modeled as a piecewise function, with different equations for various wind speed intervals. Electricity generation begins at the cut-in speed, while the wind density, blade swept area, and efficiency coefficient influence power output [63]. The turbine reaches its maximum rated power at the rated wind speed and shuts down if wind speed exceeds the cut-out speed for safety. The power law equation for wind speed at hub height is given by [40]:


$$\frac{V_{2}}{V_{1}}={\left(\frac{h_{2}}{h_{1}}\right)}^{\gamma },$$ (3)

where, $V_{1}$ is the wind speed at reference height $h_{1}$ (m/s). $V_{2}$ is the wind speed at hub height $h_{2}$ (m/s). γ is the friction coefficient, influenced by wind speed, terrain roughness, height, temperature, and time of day or year. Typical values of γ are 0.11 for extreme wind conditions and 0.2 for normal conditions per IEC 61400−1 [64]. A common practical value is 1/7. The WT generator’s power output is given by:


$$P_{WT}(t)=\left\{ \begin{array}{ccc} 0 & , & V\leq V_{cut-in}\\ V^{3}\left(\frac{P_{r}}{V_{rated}^{3}-V_{cut-in}^{3}}\right)-P_{r}\left(\frac{V_{cut-in}^{3}}{V_{rated}^{3}-V_{cut-in}^{3}}\right) & , & V_{cut-in}<V<V_{rated}\\ P_{r} & , & V_{rated}\leq V\leq V_{cut-out}\\ 0 & , & V\geq V_{cut-out} \end{array},\right.\;$$ (4)

where, $P_{r}$ is the rated power (kW). V is the wind speed (m/s). $V_{cut-in}$, $V_{rated}$, and $V_{cut-out}$ are the cut-in, rated, and cut-out wind speeds (m/s), respectively.

The rated power of the WT generator is also expressed as:


$$P_{r}=\frac{1}{2}C_{p}\times \rho_{air}\times A_{wind}\times V_{rated}^{3}$$ (5)

where, $C_{p}$ is the maximum power coefficient. $\rho_{air}$ is air density (kg/m³). $A_{wind}$ is the blade swept area (m²).

#### 2.2.2 Battery storage system (BSS) modeling.

Renewable energy sources (RESs) in microgrid (MG) systems experience fluctuations due to environmental conditions. Energy storage systems (ESSs), particularly battery banks, help stabilize these variations and ensure a reliable power supply. The battery bank acts as a buffer, storing excess energy generated during peak production and supplying it when RES output is insufficient. This balance reduces the risks associated with unpredictable power generation. In most reported cases study in literature, where RESs cannot meet the load demand, alternative power sources, such as diesel generators, may be used. However, integrating battery storage minimizes reliance on non-renewable backups, enhancing system sustainability and resilience. In this study the DG has not been included in any configuration of the study. The state of charge (SoC) of the BSS is determined based on charging and discharging conditions [64]:

**Charging Mode:**


$$SoC(t+\Delta t)=SoC(t)\times (1-\sigma_{b})+\left(P_{PV}\;(t)\;\times \;\eta_{inv}\;+\frac{P_{WT}(t)}{\eta_{inv}}-\;\frac{P_{L}(t)}{\eta_{inv}}\right)\times \eta_{BC}\times \Delta t$$ (6)

**Discharging Mode:**


$$SoC(t+\Delta t)=SoC(t)\times (1-\sigma_{b})-\left(\frac{P_{L}(t)}{\eta_{BD}}\;-P_{WT}(t)-\;P_{PV}\;(t)\;\times \;\eta_{inv}\right)\times \eta_{BD}\times \Delta t$$ (7)

where, $P_{L}(t)$ is the electrical load demand (kW). $P_{PV}(t)$ is the PV output power (kW). $\eta_{inv}$ is the inverter efficiency (%). $\sigma_{b}$ is the BSS self-discharge rate (%). $\eta_{C}$ and $\eta_{BD}$ are the charging and discharging efficiencies of the BSS (%), respectively. To ensure the longevity of the ESS, the SoC must remain within the defined operational limits:


$${\text{SoC}}_{\min}\leq \text{SoC}\leq {\text{SoC}}_{\max}.$$ (8)

#### 2.2.3 Electrolyzer.

Water electrolysis is an electrochemical process that splits water molecules into hydrogen and oxygen gases. This occurs in an electrolyzer cell comprising two electrodes: a cathode and an anode. At the cathode, hydrogen gas (H₂) is produced through the reduction reaction, while at the anode, oxygen gas (O₂) is generated via oxidation. These reactions are represented as follows [65]:

Cathode reaction:


$$2H_{2}O+2e^{-}\to H_{2}+2OH^{-}$$ (9)

Anode reaction:


$$2OH^{-}\to O_{2}+2H_{2}O+4e^{-}$$ (10)

The efficiency of water electrolysis depends on several factors, including electrolyte composition, electrode materials, applied voltage, and temperature. Understanding these parameters is essential for optimizing hydrogen production technologies, particularly for renewable energy storage applications.

Electrolysis is a well-established method for hydrogen generation, especially when powered by renewable electricity, due to its high energy efficiency. Alkaline and Proton Exchange Membrane (PEM) electrolyzers are the two most widely used types [66]. The power consumption of an electrolyzer is given by:


$$P_{EL}=\frac{\dot{m_{H_{2}}}\cdot HHV_{H_{2}}}{\eta_{EL}}$$ (11)

where, $P_{EL}$ is the power consumption (kW). $\dot{m_{H_{2}}}$ is the hydrogen flow rate (kg/s). $HHV_{H_{2}}$ is the higher heating value of hydrogen (MJ/kg). $\eta_{EL}$ is the electrolyzer efficiency.

In this study, a generic electrolyzer with an operational efficiency of 85% and a lifespan of 15 years was used. The explored electrolyzer capacities were 10 kW, 20 kW, and 50 kW, chosen to evaluate performance across different operational scales. This approach ensures a comprehensive assessment of electrolysis efficiency and viability for hydrogen production applications.

#### 2.2.4 Fuel cell.

Fuel cells perform the reverse process of electrolysis, converting hydrogen into electricity through electrochemical reactions. A fuel cell consists of an anode and a cathode, where hydrogen oxidation occurs at the anode, releasing protons and electrons [67]:

**Anode reaction:**


$$H_{2}\to 2H^{+}+2e^{-}$$ (12)

**Cathode reaction:**


$$O_{2}+4e^{-}+4H^{+}\to 2H_{2}O$$ (13)

The electrical output of a fuel cell depends on the voltage generated by these reactions, which is expressed as:


$$V_{FC}=E_{rev}-V_{act}-V_{diff}-V_{\Omega }$$ (14)

where, $E_{rev}$ is the reversible voltage. $V_{act}$ is the activation voltage loss. $V_{diff}$ is the diffusion voltage loss. $V_{\Omega }$ is the ohmic voltage loss.

Fuel cells are efficient and environmentally friendly energy conversion devices used in transportation, stationary power generation, and backup power systems. This study employed a standardized fuel cell with a nominal operational lifespan of 50,000 hours. The examined power capacities ranged from 5 kW to 20 kW in 5 kW increments, ensuring a comprehensive performance analysis across various output levels.

#### 2.2.5 Hydrogen tank.

A hydrogen storage tank is crucial for managing surplus hydrogen generated by the electrolyzer during periods of high renewable energy production. This stored hydrogen is later utilized when renewable generation is insufficient to meet load demand. A key assumption in this study is that the tank experiences no hydrogen leakage, ensuring the integrity of the stored supply. Additionally, an initial hydrogen quantity equivalent to 10% of the tank’s total volume was considered at the start of operations. The storage capacity was explored within a defined range of 50 kg, 100 kg, and 150 kg to assess storage requirements under varying hydrogen production and demand conditions.

#### 2.2.6 Converter modeling.

Converters enable energy flow between different components in microgrid (MG) systems. They operate as inverters, converting direct current (DC) to alternating current (AC), and as rectifiers, transforming AC to DC. In hybrid systems, they ensure seamless energy exchange between DC and AC sections. The inverter’s capacity, $P_{inv}$, is determined by the following equations:


$$P_{inv}=\frac{E_{L}(\max)}{\eta_{\frac{dc}{ac}}},$$ (15)

$$\eta_{\frac{dc}{ac}}\left[P_{\text{BSS,ch/dis}}\;(t)\;+\;P_{\text{PV}}\;(t)\right]\leq P_{\text{inv}},$$ (16)

$$\eta_{\frac{ac}{dc}}\left[P_{G}\;(t)\right]\leq P_{\text{inv}},$$ (17)

where, $P_{inv}$ is the converter capacity (kW). $E_{L}(\max)$ is the maximum electrical load demand (Wh). $\eta_{\frac{dc}{ac}}$ and $\eta_{\frac{ac}{dc}}$ are the efficiencies for DC-to-AC and AC-to-DC conversion, respectively. $P_{BSS,ch/dis}$ represents the charge/discharge power of the battery storage system (BSS). $P_{PV}$ is the PV power output. $P_{G}$ denotes the imported/exported grid power.

### 2.3 HOMER simulation tool

This study aims to design an economically optimized standalone HRES while ensuring compliance with operational constraints. The total annual cost encompasses the initial investment, operation, and maintenance (O&M) costs for each power source, and the salvage value of the equipment, which is deducted from the overall cost. The procedure of the optimal configuration using HOMER software has been thoroughly examined [68].

The primary goal of using HOMER is to model and optimize the proposed hybrid energy system’s technical and economic performance. HOMER’s optimization algorithms allow for the simulation of various system configurations under different scenarios, considering factors such as renewable energy generation profiles, energy storage capabilities, and load demand. By utilizing HOMER, we can determine the most cost-effective and reliable system configuration, balancing the energy production from renewable sources with the storage and backup power needs. In this study, HOMER was used to determine the optimal configuration for the hybrid system by performing the following steps:

- System Modeling: The energy resources (solar, wind, and battery storage) and load profiles were input into HOMER, along with relevant system constraints, such as equipment capacities, maintenance schedules, and operational limits.
- Optimization Process: HOMER’s optimization engine was used to evaluate various configurations, selecting the one that minimizes the total Net Present Cost (NPC) while meeting the required energy demand. The software also took into account factors like energy storage charging/discharging cycles and the integration of grid power when necessary.
- Sensitivity and Scenario Analysis: A series of sensitivity analyses were conducted to understand the effect of different variables, such as changes in resource availability or fuel prices, on the system’s performance. This helped identify the most robust system design under various operational conditions.

The use of HOMER in similar studies is well-documented in the literature. For instance, Authors in [54] used HOMER for optimizing a hybrid energy system involving solar PV, wind, and battery storage, achieving a significant reduction in system costs. By using HOMER’s optimization capabilities, this study ensures that the proposed hybrid energy system is not only technically feasible but also economically viable, providing a robust solution to meet the energy demands of the selected region.

The Net Present Cost (NPC) serves as the primary economic metric in HOMER Pro, assessing the total system cost over its operational lifetime [69]. This includes capital, replacement, operation and maintenance (O&M), and other expenses, while deducting any revenue generated. NPC is determined as follows [68]:


$$\begin{array}{c} NPC=\sum_{t=1}^{T}\frac{C_{t}-R_{t}}{(1+r{)}^{t}} \end{array}$$ (18)

where, $C_{t}$ represents cash outflows in year t, covering capital expenditures, replacements, O&M, and other costs. $R_{t}$ denotes cash inflows in year t, including revenues or cost savings. T is the system’s total analysis period (25 years). r is the real discount rate, reflecting the time value of money and inflation.

The real discount rate (r) incorporates inflation effects and is derived from the nominal discount rate ($i^{\prime }$) of 5.68% and the expected inflation rate (f) of 4.2%, as follows [69]:


$$r=\frac{i^{\prime }-f}{1+f}$$ (19)

To determine the Levelized Cost of Energy (LCOE), it is necessary to first compute the Total Annualized Cost ($C_{\text{ann,tot}}$) and the Capital Recovery Factor (CRF), which converts lifetime system costs into annualized values for consistent comparisons. The analysis assumes a 25-year system lifespan. The Total Annualized Cost is obtained as the following:


$$C_{\text{ann,tot}}=CRF(r,T)\times NPC$$ (20)

where the Capital Recovery Factor is expressed as:


$$CRF(r,T)=\frac{r(1+r{)}^{T}}{(1+r{)}^{T}-1}$$ (21)

**Fig 3:**
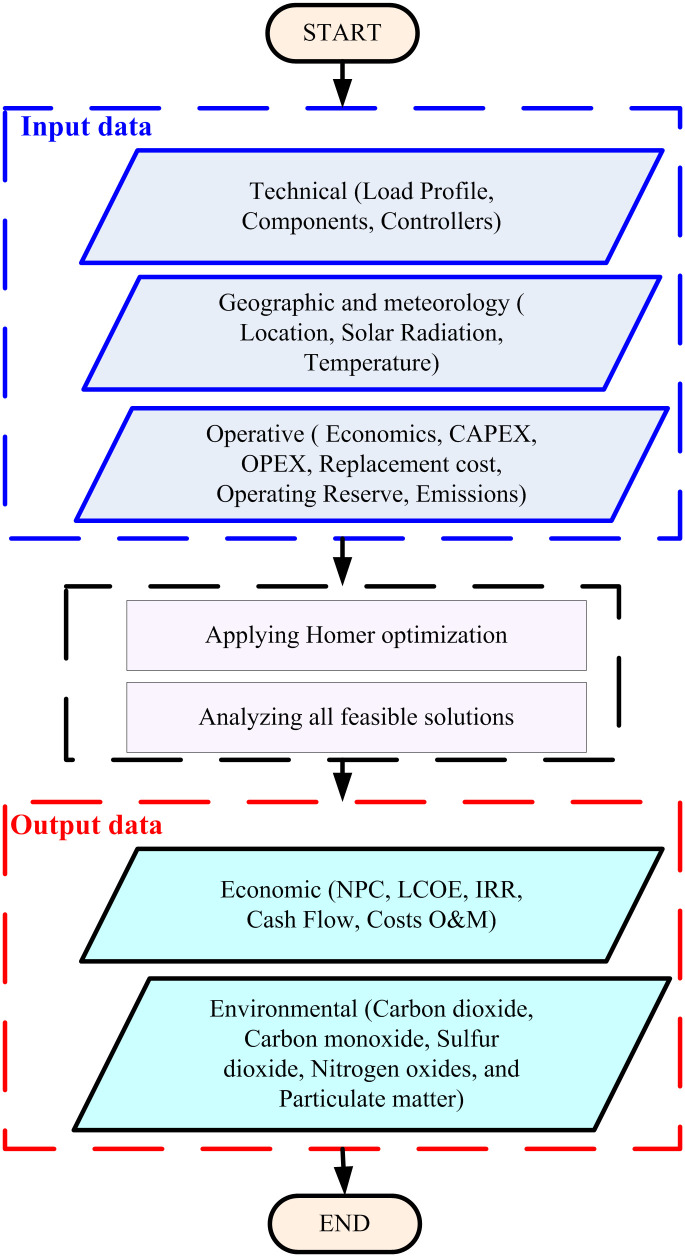
Framework of Input and Output Data for HRES Optimization in HOMER.

After calculating $C_{\text{ann,tot}}$, the LCOE is determined as follows:


$$LCOE=\frac{C_{\text{ann,tot}}}{E_{\text{served}}}$$ (22)

where $E_{\text{served}}$ represents the total energy supplied by the system throughout its operational life, including energy delivered to primary and deferrable loads as well as energy sold to the grid. Fig 3 illustrates the overall framework of the input and output data necessary for effective HRES optimization in HOMER.

### 2.4 Standalone HRES components

The detailed specifications are summarized based on relevant studies in Table 2 [34,37]. There is an assumption which related to the electrolyzer cost, due to oxidation effects from the acidic content in water, desalination must be implemented before introducing water into the electrolyzer to prevent component degradation. The cost of desalination is negligible compared to overall system expenses, making it a viable addition without significantly affecting economic feasibility [54]. This process is essential for optimizing hydrogen production and ensuring the reliability of hybrid renewable energy systems. While desalination was assumed negligible in this study, its necessity depends on the water source. The project lifetime is set to 25 years. Moreover, the maximum allowable annual capacity shortage is limited to 5% of the total electrical load.

**Table 2:** Technical specifications and study assumptions.

Description	Data
**PV**	
Capital cost	1000 US $/ kW
Lifetime	25 years
Operation and maintenance cost	10 US $/kW/year
**Alkaline electrolyzer**	
Efficiency	85%
Lifetime	15 years
Initial cost	2000 US$/kW
Replacement cost	1400 US$/kW
O & M cost	10 US$/kW/year
**Fuel Cell**	
Initial cost	3000 US$/kW
Replacement cost	2700 US$/kW
O & M cost	0.01 US$/operation h
Lifetime	15,000 h
**Inverter**	
Capital	500 US $/kW
Lifetime	15 years
Operation and maintenance cost	0 US $/year
**Wind**	
Capital cost	900 US $/kW
Lifetime	25 years
Operation and maintenance cost	36 US $/kW/year
**H2 storage tank**	
Lifetime	25 years
Initial cost	800 US$/kg
Replacement cost	700 US$/kg
O & M cost	3 US$/kg/year
**Batteries**	
Type of batteries	Polarium SLB48-200-146-2
Nominal voltage (V)	50.8 V
Nominal capacity (kWh)	10.2
Nominal capacity (Ah)	200
Minimum State of charge	20%
Roundtrip efficiency	90%
Operation and maintenance	2$/year
Cost	1150 $
Lifetime	15 years

### 2.5 Site data

**Fig 4:**
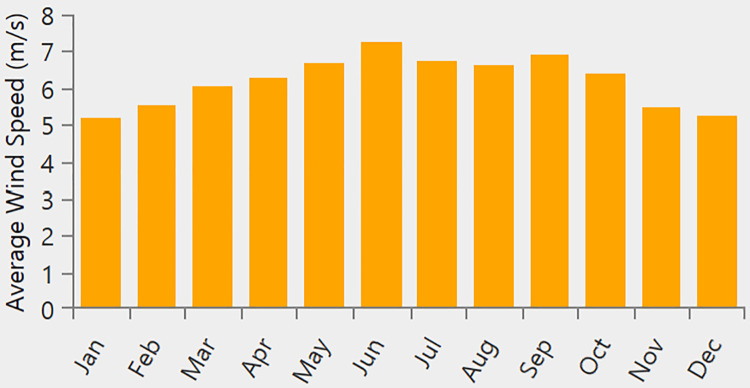
Average Wind speed at the tested location.

**Fig 5:**
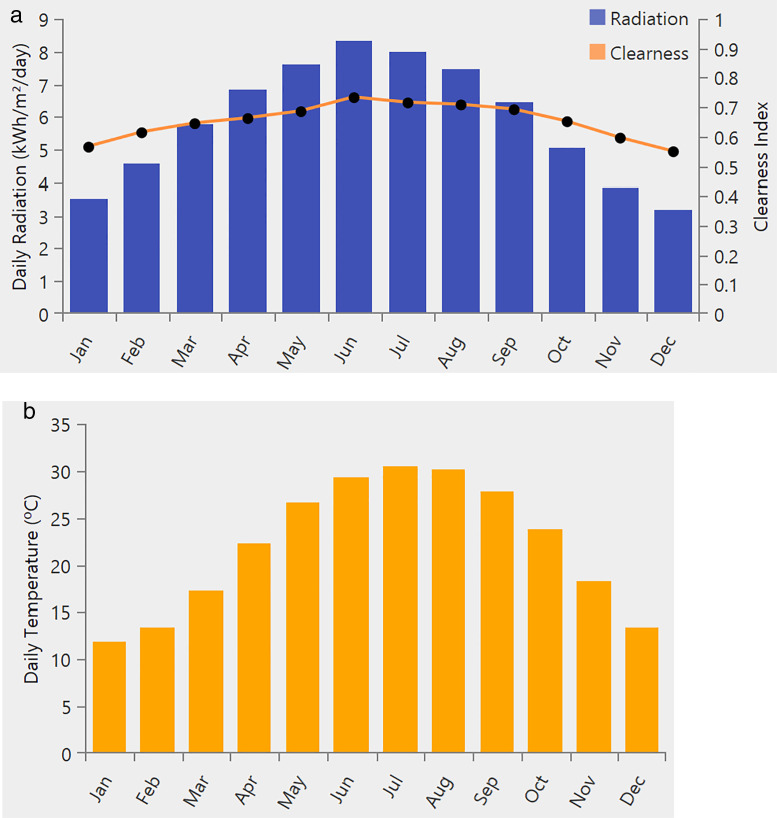
a) Daily radiation at the tested location and b) The daily temperature at the tested location.

**Fig 6:**
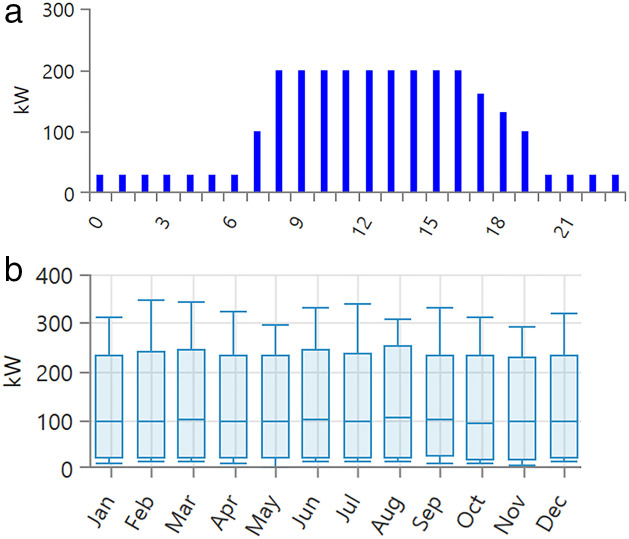
a) Daily profile of the load demand and b) seasonal load profile kW.

Fig 4 presents the site-specific wind speed data for a location in Neom, Saudi Arabia (Location: 28°7.8’N, 35°48.4’E), with an average annual wind speed of 4.9 m/s. Moreover, Fig 5a illustrates the solar radiation data, where the average annual global horizontal irradiance (GHI) is measured at 5.88 kWh/m²/day. Fig 5b shows the annual temperature variations, with an average recorded temperature of 22.04°C. The load profile is illustrated in Fig 6, with Fig 6a detailing the daily commercial load demand and Fig 6b presenting the seasonal load profile in kilowatts (kW). The peak load reaches 347.79 kW, while the average load is 100.93 kW. The average annual load consumption is calculated at 2422.3 kWh/day.

## 3. Simulation results

### 3.1 Diesel generator

To clear the motivation of the paper, a case study of feeding the load demand with diesel generator (DG) only. The results of the DG system analysis show a system designed to meet a load following dispatch strategy with a generator size of 390 kW. The system’s net present cost is $15.9 million, which includes $331,500 in capital costs, $2.56 million in operating costs, and $4.64 million in replacement costs. The fuel resource cost is significant at $8.51 million, while a salvage value of -$132,600 brings the overall financial picture into focus. The levelized cost of energy (LCOE) remains at $0.720 per kWh.

The electricity production from the DG system amounts to 1,169,307 kWh annually, which is more than enough to meet the primary load demand of 884,165 kWh per year. The system generates an excess of 285,142 kWh annually, which could potentially be used for additional loads or stored for later use. There is no unmet load in the system, indicating that the generator is capable of fully supplying the demand. However, a small capacity shortage of 13.1 kWh per year suggests that, while the system is generally reliable, it may face brief periods of underperformance when demand peaks.

Fuel consumption statistics indicate that the system uses 340,242 liters of diesel annually, with an average fuel consumption of 932 liters per day and 38.8 liters per hour. These figures provide insight into the operational fuel needs of the system and underscore the environmental impact, especially in terms of emissions. The system produces significant carbon emissions, with 890,622 kg of CO₂ emitted per year, along with other pollutants, including carbon monoxide (5,614 kg/yr), unburned hydrocarbons (245 kg/yr), particulate matter (34 kg/yr), sulfur dioxide (2,181 kg/yr), and nitrogen oxides (5,274 kg/yr).

When comparing the base system with the proposed system, no improvements in net present cost, capital, operating expenses, or emissions are observed. The levelized cost of energy (LCOE) remains at $0.720 per kWh for both systems. This suggests that, in its current form, the system does not achieve any operational or environmental benefits over the base design. The results indicate that further optimization efforts are needed, whether through improving fuel efficiency, integrating renewable energy sources, or adopting newer, more efficient technologies to lower costs and emissions.

### 3.2 Full renewable energy systems

#### Configuration 1: PV/BSS system.

The microgrid system consists of photovoltaic (PV) panels, lithium-ion battery storage, and a system converter. The PV system has a capacity of 936 kW, supported by 1,120 strings of 1 kWh lithium-ion batteries for energy storage. The system converter has a capacity of 402 kW, and the dispatch strategy follows the HOMER Load Following method to optimize energy use.

The total net present cost (NPC) of the system is $2.42M, with a capital expenditure (CAPEX) of $1.39M and annual operating expenses (OPEX) of $41,477. The levelized cost of electricity (LCOE) is $0.112/kWh, slightly higher than the base system. The system remains carbon-neutral with zero fuel consumption. The cost breakdown highlights major investments in PV panels, battery storage, and the system converter, ensuring long-term operational efficiency. The results have been listed in Table 3.

**Table 3:** Net Present Costs of PV/BSS system.

Parameter	Capital	Operating	Replacement	Salvage	Total
**Battery**	$616,000	$280,000	$616,000	($200,441)	$1.31M
**PV**	$608,532	$234,051	$0.00	$0.00	$842,582
**Converter**	$160,969	$0.00	$160,969	($53,656)	$268,282
**System**	$1.39M	$514,051	$776,969	($254,097)	$2.42M

The system generates a total of 1,842,482 kWh per year, entirely from PV panels. The primary load consumption is 865,456 kWh per year, covering 100% of the total demand. However, excess electricity production reaches 908,213 kWh per year, indicating potential for improved utilization. The system also experiences an unmet electric load of 18,709 kWh per year and a capacity shortage of 44,496 kWh per year, signaling areas that need optimization.

The system effectively meets most of the demand, but the high excess electricity output suggests that additional energy storage or alternative utilization strategies could improve efficiency. The increased unmet load and capacity shortages compared to the previous configuration indicate that further refinement in dispatch strategies and system sizing is necessary for enhanced performance.

**Fig 7:**
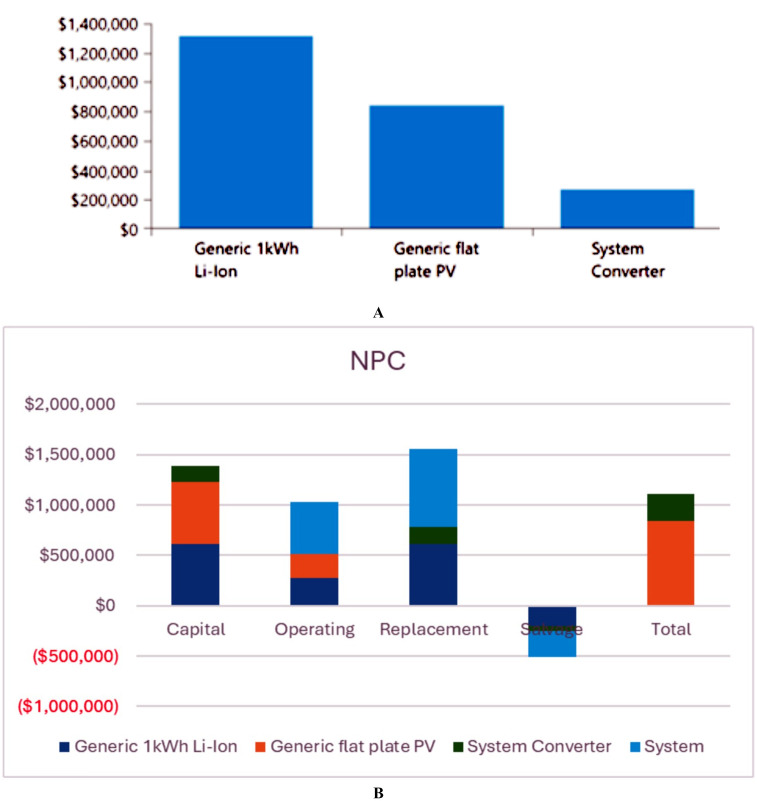
Net present value (NPV) for each system component for the PV/BSS system; a) total NPV and b) components breakdown costs.

**Fig 8:**
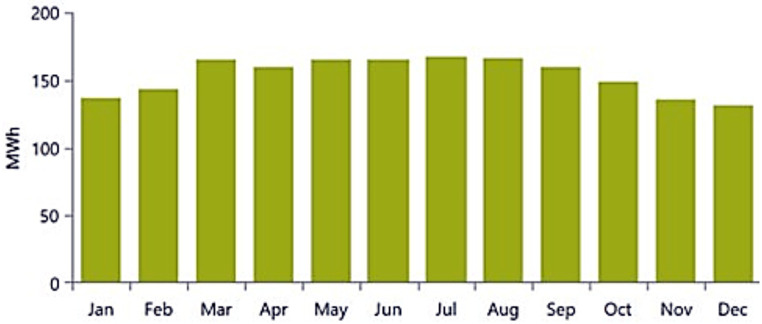
Average monthly electric energy generation from renewable sources in the first configuration PV/BSS.

**Fig 9:**
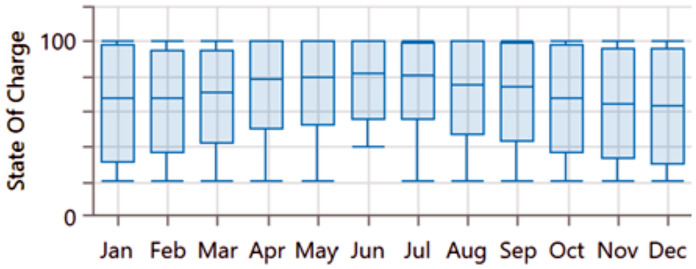
Average monthly state of charge for the first configuration PV/BSS.

**Fig 10:**
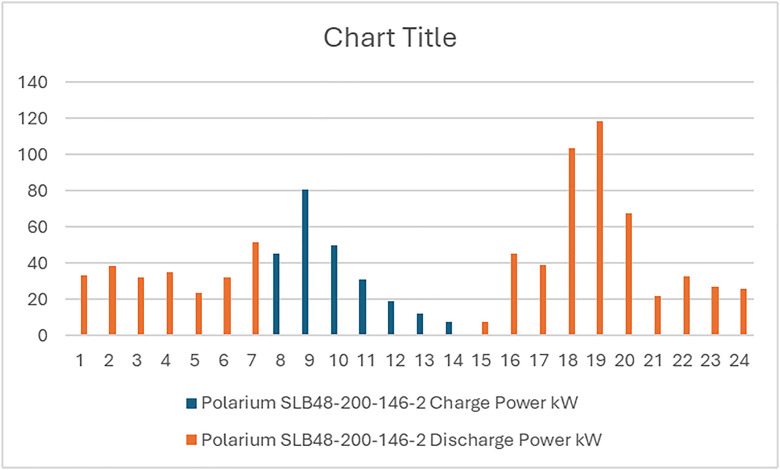
BSS charging and discharging for two days for the first configuration PV/BSS.

**Fig 11:**
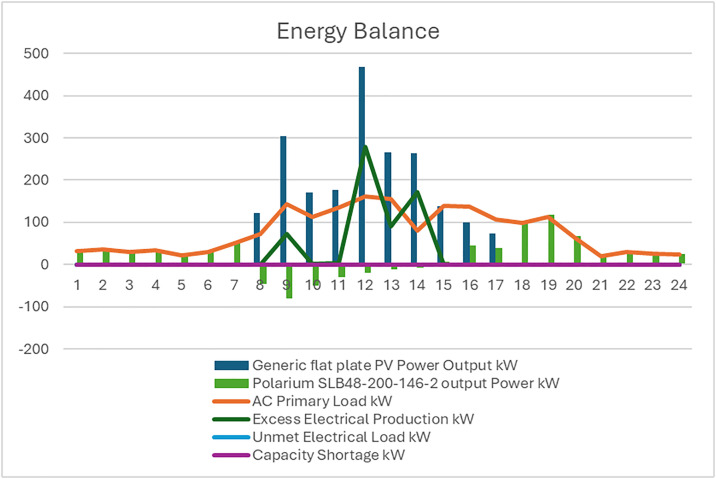
The energy balance of the first configuration for one day PV/BSS.

The analysis of the PV/BSS system includes various performance metrics, with Fig 7 illustrating the net present value (NPV) for each system component. Specifically, Fig 7a shows the total NPV, while Fig 7b provides a breakdown of the component costs. Fig 8 presents the average monthly electric energy generation from renewable sources in the first configuration, emphasizing the system’s production patterns. Fig 9 illustrates the average monthly state of charge, reflecting the battery storage system’s performance throughout the month. The charging and discharging patterns of the battery storage system over two days are shown in Fig 10, which highlights the system’s operation during periods of excess and low energy generation. Finally, Fig 11 provides an energy balance for a single day, showcasing the interactions between the PV panels and the battery storage system in maintaining energy supply.

#### Configuration 2: PV//WT/BSS system.

The microgrid system consists of a combination of photovoltaic (PV) panels, wind turbines, lithium-ion battery storage, and a system converter. The PV system has a capacity of 783 kW, supported by 254 wind turbines rated at 1 kW each. The energy storage consists of 831 strings of 1 kWh lithium-ion batteries, ensuring reliability and backup power. The system converter has a capacity of 279 kW, and the dispatch strategy follows the HOMER Load Following method to optimize energy use.

The total net present cost (NPC) of the system is $2.11M, with a capital expenditure (CAPEX) of $1.24M and annual operating expenses (OPEX) of $34,485. The levelized cost of electricity (LCOE) remains at $0.0968/kWh, making the system financially viable. Notably, the system achieves zero carbon emissions and does not rely on fuel consumption, highlighting its sustainability. The breakdown of costs includes significant investments in PV panels, battery storage, and system converters, ensuring a balance between capital costs and long-term operational efficiency. Table 4 listed the NPC for PV//WT/BSS system

**Table 4:** Net Present Costs for PV//WT/BSS system.

Parameter	Capital	Operating	Replacement	Salvage	Total
WT	$135,000	$95,875	$0.00	$0.00	$230,875
PV	$448,382	$57,318	$0.00	$0.00	$505,700
Battery	$193,200	$0.00	$80,616	-$15,005	$258,810
Converter	$130,008	$0.00	$54,248	-$10,097	$174,159
System	$906,590	$153,193	$134,864	-$25,102	$1.17M

The system generates a total of 1,887,481 kWh per year, with 81.7% of this coming from PV panels (1,541,301 kWh) and the remaining 18.3% from wind turbines (346,180 kWh). The primary load consumption is 869,658 kWh per year, accounting for 100% of the total demand. However, there is a substantial amount of excess electricity at 968,965 kWh per year, indicating overproduction relative to demand. The system experiences an unmet electric load of 14,507 kWh per year and a capacity shortage of 45,027 kWh per year, suggesting areas for optimization.

While the system is effective in meeting most of the demand, the high excess electricity production indicates an opportunity for improved energy storage or alternative utilization strategies, such as integrating additional loads or selling excess power. The presence of unmet electric load and capacity shortages suggests that adjustments in dispatch strategies and component sizing could enhance overall performance. Utilizing excess electricity for additional loads, hydrogen production, or grid export could enhance system utilization and economic feasibility.

**Fig 12:**
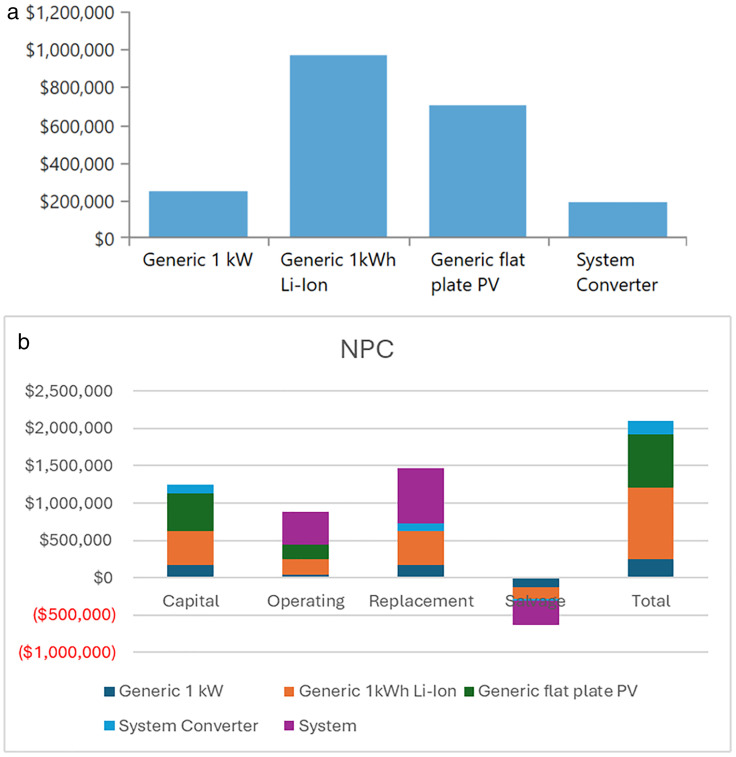
Net present value (NPV) for each system component for the PV/WT/BSS system; a) total NPV and b) components breakdown costs.

**Fig 13:**
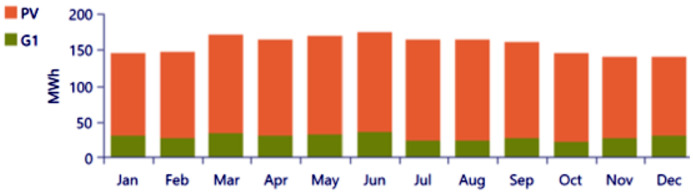
Average monthly electric energy generation from renewable sources in the second configuration PV/WT/BSS.

**Fig 14:**
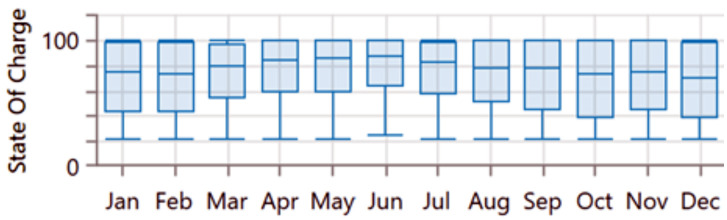
Average monthly state of charge for the second configuration PV/WT/BSS.

**Fig 15:**
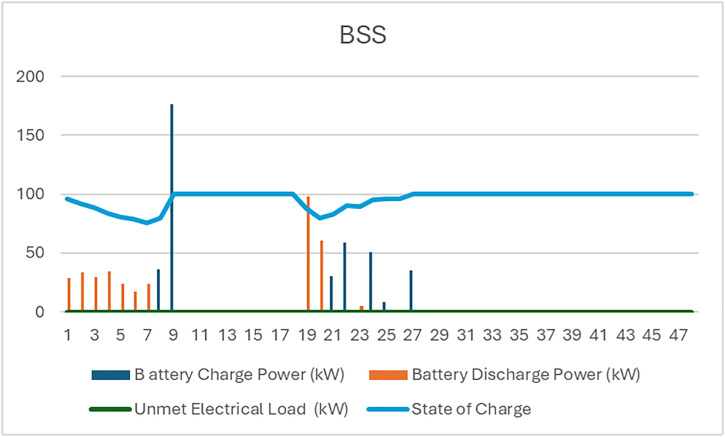
BSS charging and discharging for two days for the second configuration PV/WT/BSS.

**Fig 16:**
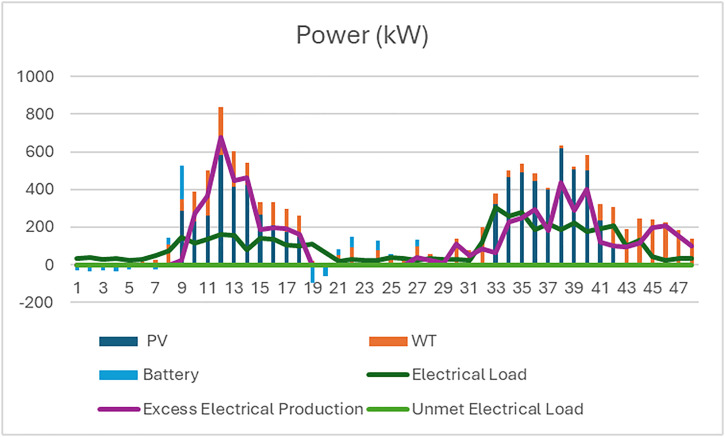
The energy balance of the second configuration for one day PV/WT/BSS.

The analysis of the PV/WT/BSS system is presented through various performance metrics. Fig 12 shows the net present value (NPV) for each system component. Specifically, Fig 12a illustrates the total NPV, while Fig 12b provides a breakdown of the component costs. Fig 13 displays the average monthly electric energy generation from renewable sources in the second configuration, highlighting the system’s production from both PV and wind sources. Fig 14 shows the average monthly state of charge, reflecting the performance of the battery storage system throughout the month. The BSS charging and discharging patterns over two days are depicted in Fig 15, demonstrating how the system manages excess and low energy periods. Lastly, Fig 16 presents the energy balance for one day, illustrating the interaction between PV panels, wind turbines, and the battery storage system.

#### Configuration 3: PV//WT/BSS/ hydrogen tank/ FC/ electrolyzer system.

In this scenario, the renewable energy system is designed to meet both the load demand and the energy requirements of the electrolyzer and battery system. The microgrid system consists of several key components, designed to meet the energy needs through a mix of renewable sources and storage. The major components include a 50 kW Fuel Cell Generator, a 909 kW flat plate PV system, 631 strings of Li-Ion Battery Storage, 303 wind turbines, a 283 kW system converter, a 50 kW electrolyzer, and a 50 kg hydrogen tank. The dispatch strategy used is HOMER Load Following. The Net Present Cost (NPC) is $2.30M in the proposed system. The Capital Expenditure (CAPEX) is $1.54M. The Levelized Cost of Electricity (LCOE) is from $0.106/kWh due to the higher capital investment in the proposed system. Table 5 listed the results for such system.

**Table 5:** Net Present Costs for PV/WT/BSS/Hydrogen Tank/FC/Electrolyzer system.

Parameter	Capital	Operating	Replacement	Salvage	Total
**Fuel Cell**	$150,000.00	$0.00	$11,125.00	($59,906.25)	$101,218.75
**WT**	$196,950.00	$196,950.00	$45,450.00	($147,712.50)	$291,637.50
**Battery**	$347,050.00	$347,050.00	$157,750.00	($115,683.33)	$736,166.67
**Electrolyzer**	$100,000.00	$70,000.00	$12,500.00	($23,333.33)	$159,166.67
**PV**	$590,606.25	$0.00	$227,156.25	$0.00	$817,762.50
**H2 Tank**	$40,000.00	$0.00	$0.00	$0.00	$40,000.00
**Converter**	$113,100.00	$113,100.00	$0.00	($37,700.00)	$188,500.00
**System**	$1,537,706.25	$727,100.00	$453,981.25	($384,335.42)	$2,334,452.08

Moreover, the system generates a total of 2,219,942 kWh/year. The largest share of this comes from the PV system, which accounts for 80.6% of the total production, or 1,788,208 kWh/year. The wind turbines contribute 18.6%, producing 412,962 kWh/year, while the fuel cells provide a small fraction, 0.846%, or 18,771 kWh/year. A significant portion of the generated energy, 1,124,275 kWh/year (50.6%), is excess electricity, indicating inefficiencies in the energy utilization and possible overproduction relative to consumption.

The system’s total electricity consumption stands at 1,050,675 kWh/year. The AC primary load is the largest consumer, accounting for 82.7% of the total at 868,910 kWh/year. The electrolyzer consumes 17.3%, or 181,765 kWh/year, while there are no DC or deferrable loads in the system. The absence of such loads further limits the system’s flexibility in energy consumption, and the high level of excess electricity suggests that storage and load management strategies should be revisited. It is noted that the system still faces an unmet electric load of 1.73%

The largest capital investment in both systems is in the PV panels, totaling $590,606 for the proposed system, followed by battery storage and wind turbines. Although the proposed system reduces operating costs, the introduction of fuel consumption affects its overall efficiency. Improving the integration of hydrogen production based the excess energy could enhance the system’s efficiency.

**Fig 17:**
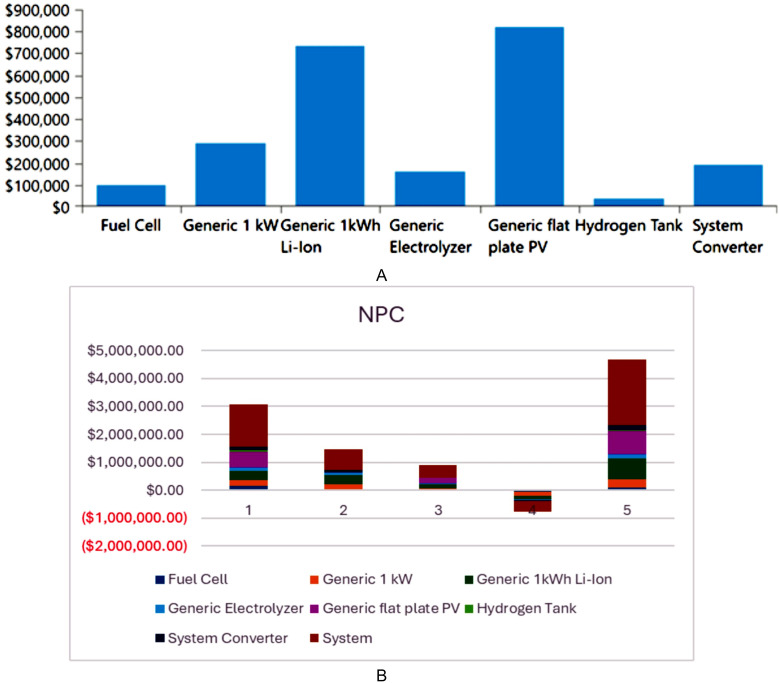
Net present value (NPV) for each system component for the PV//WT/BSS/ Hydrogen Tank/ FC/ Electrolyzer system; a) total NPV and b) Components breakdown costs.

**Fig 18:**
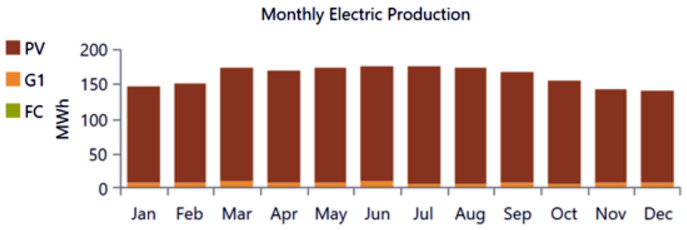
Average monthly electric energy generation from renewable sources in the third configuration PV//WT/BSS/ Hydrogen Tank/ FC/ Electrolyzer.

**Fig 19:**
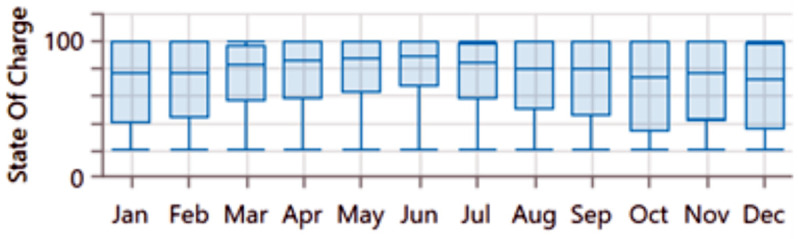
Average monthly state of charge for the third configuration PV//WT/BSS/ Hydrogen Tank/ FC/ Electrolyzer.

**Fig 20:**
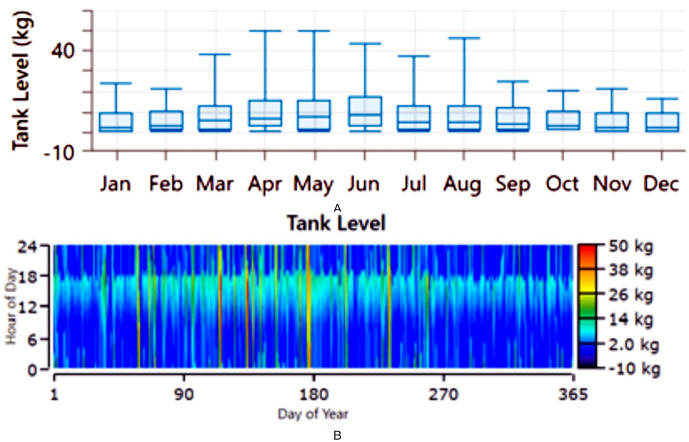
a) Average monthly Tank Level for the third configuration PV//WT/BSS/ Hydrogen Tank/ FC/ Electrolyzer, b) Tank level.

**Fig 21:**
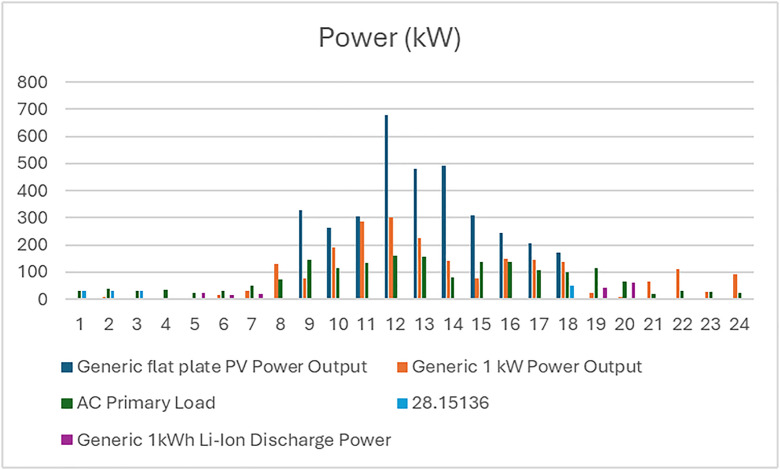
The energy balance of the third configuration for one day PV//WT/BSS/ Hydrogen Tank/ FC/ Electrolyzer.

The performance of the PV//WT/BSS/Hydrogen Tank/FC/Electrolyzer system is analyzed through various metrics. Fig 17 presents the net present value (NPV) for each system component. Specifically, Fig 17a shows the total NPV, while Fig 17b provides a breakdown of the component costs. Fig 18 illustrates the average monthly electric energy generation from renewable sources in the third configuration, highlighting contributions from PV, wind, and hydrogen. Fig 19 presents the average monthly state of charge, demonstrating the performance of the battery storage system throughout the month. Fig 20 showcases the average monthly tank level for the hydrogen storage system, with Fig 20a representing the tank level across the month and Fig 20b focusing on more detailed variations. Lastly, Fig 21 provides the energy balance for one day, emphasizing the interaction between PV panels, wind turbines, the battery storage system, and the hydrogen tank.

### 3.3 Comprehensive comparison

As far as the system’s base distributed generation (DG) and its environmental impact are concerned, this section compares the various renewable energy system (RES) configurations. The three configurations of PV/BSS, PV/WT/BSS, and PV/WT/BSS/Elec/FC HRESs differ in complexity and resource integration to meet energy demands. Table 6 listed the comparison keys among the three configurations. The PV/BSS system relies on photovoltaic panels paired with lithium-ion battery storage and a system converter. With a PV capacity of 936 kW and 1,120 strings of 1 kWh batteries, the system generates a total of 1,842,482 kWh annually, entirely from the PV panels. The load consumption is 865,456 kWh, fulfilling 100% of the demand. However, the system experiences high excess electricity (908,213 kWh) and faces an unmet load of 18,709 kWh, coupled with a capacity shortage of 44,496 kWh, signaling inefficiencies in storage and load management. While the system is carbon-neutral with zero fuel consumption, the excess energy suggests a need for improved storage or utilization strategies to optimize the system’s performance. The cost breakdown shows a total NPC of $2.42M, with a CAPEX of $1.39M and an LCOE of $0.112/kWh, which is slightly higher than the other configurations.

**Table 6:** Key parameters of the three configurations.

Parameter	Configuration 1: PV/BSS	Configuration 2: PV//WT/BSS	Configuration 3: PV//WT/BSS/Hydrogen
**System Components**	PV, BSS, System Converter	PV, Wind Turbines, BSS, System Converter	PV, Wind Turbines, BSS, System Converter, Hydrogen Tank, Fuel Cell, Electrolyzer
**PV**	936 kW	783 kW	909 kW
**WT (1 kW each)**	–	254 turbines	303 turbines
**Energy Storage**	1,120 strings of 1 kWh batteries	831 strings of 1 kWh batteries	631 strings of 1 kWh batteries
**System Converter**	402 kW	279 kW	283 kW
**Fuel Cell**	–	–	50 kW
**Electrolyzer**	–	–	50 kW
**Hydrogen Tank**	–	–	50 kg
**Net Present Cost (NPC)**	$2.42M	$2.11M	$2.30M
**Capital Expenditure (CAPEX)**	$1.39M	$1.24M	$1.54M
**Levelized Cost of Electricity (LCOE)**	$0.112/kWh	$0.0968/kWh	$0.106/kWh
**Total Energy Generated**	1,842,482 kWh/year	1,887,481 kWh/year	2,219,942 kWh/year
**PV Contribution**	100% (1,842,482 kWh)	81.7% (1,541,301 kWh)	80.6% (1,788,208 kWh)
**Wind Contribution**	–	18.3% (346,180 kWh)	18.6% (412,962 kWh)
**Fuel Cell Contribution**	–	–	0.846% (18,771 kWh)
**Primary Load Consumption**	865,456 kWh/year	869,658 kWh/year	868,910 kWh/year
**Excess Energy**	908,213 kWh/year	968,965 kWh/year	1,124,275 kWh/year
**Unmet Load**	18,709 kWh/year	14,507 kWh/year	15,255 kWh/year
**Capacity Shortage**	44,496 kWh/year	45,027 kWh/year	44,799 kWh/year
**CO2 Emissions**	Zero	Zero	Zero

The PV//WT/BSS system builds on the PV/BSS setup by incorporating 254 wind turbines, adding 346,180 kWh to the total generation, and diversifying the renewable energy sources. The system generates 1,887,481 kWh annually, with 81.7% from PV panels and 18.3% from wind turbines. Despite meeting the primary load consumption (869,658 kWh) fully, the system still produces a significant excess (968,965 kWh), similar to Configuration 1. It also faces an unmet load of 14,507 kWh and a capacity shortage of 45,027 kWh. However, the system benefits from a lower LCOE of $0.0968/kWh, reflecting its more cost-effective nature compared to Configuration 1. The total NPC is $2.11M, with a CAPEX of $1.24M, making this configuration financially viable. The addition of wind energy reduces reliance on PV but still leaves room for optimization, particularly in handling excess electricity more efficiently, potentially through energy storage, grid export, or hydrogen production.

The PV//WT/BSS/Hydrogen configuration is the most complex of the three, integrating hydrogen storage and fuel cell technology alongside the PV and wind generation systems. With a PV capacity of 909 kW, 303 wind turbines, and 631 strings of 1 kWh batteries, this system generates a total of 2,219,942 kWh per year. PV panels contribute 80.6% of this generation, while wind turbines provide 18.6%, and fuel cells contribute a small fraction. Despite having the highest generation capacity, this system also faces significant excess energy production, with 1,124,275 kWh marked as excess. The total electricity consumption stands at 1,050,675 kWh, with a primary load of 868,910 kWh and electrolyzer consumption at 181,765 kWh. Though the system has a relatively low unmet load of 1.73%, the addition of hydrogen production, storage, and fuel cells introduces complexity and inefficiencies. The system’s NPC is $2.30M, with a CAPEX of $1.54M and an LCOE of $0.106/kWh. The complexity of integrating hydrogen production with excess energy presents opportunities for optimization but also introduces higher capital costs.

In terms of cost, Configuration 2 stands out as the most cost-effective, with the lowest NPC ($2.11M) and LCOE ($0.0968/kWh). This configuration offers a balanced approach by combining PV and wind energy, making it financially viable while meeting demand with a lower unmet load compared to Configuration 1. Configuration 1, while effective in using solar power, suffers from high excess energy and unmet load, leading to inefficiencies in utilization. Configuration 3, though technologically advanced with the integration of hydrogen, faces the challenge of high excess energy and increased capital costs, making it less cost-effective than Configuration 2, despite its higher generation capacity.

Each system presents distinct advantages. Configuration 1 is a simple, clean solution with excellent PV performance but needs more efficient storage and load strategies. Configuration 2 diversifies energy sources and improves cost efficiency, yet it still faces challenges in managing excess energy. Configuration 3 introduces cutting-edge hydrogen technology, potentially offering long-term benefits in energy flexibility but at a higher cost and complexity. In all cases, optimizing energy storage, adjusting load management strategies, and utilizing excess energy are critical to enhancing system performance and economic feasibility.

The results have been validated through the comparison with reported work of [59,70]. The cost of energy (COE) results for hybrid renewable systems optimized using the Cuckoo Search Algorithm (CSA) in Fotokol city show distinct trends across various load profiles [59]. For high and medium load conditions (HC, MC), the PV/WT/BAT/DSL system offers the lowest COE values, reaching $0.158/kWh and $0.196/kWh, respectively, with substantial CO₂ emission reductions of up to 12,444.3 kg/year. Under low load conditions (LC), the PV/BAT/DSL system outperforms others, offering a lower COE of $0.220/kWh compared to WT/BAT/DSL and other configurations. Systems integrating fuel cells (FC) exhibit extremely high COE values, exceeding $1.0/kWh in most scenarios, due to the significant capital cost of FC components (up to $60,060). The diesel-only system remains the least sustainable, with the highest COE reaching $0.766/kWh and CO₂ emissions peaking at 13,694.3 kg/year. In contrast, hybrid configurations with PV and WT achieve better cost-effectiveness and environmental benefits, making them more suitable for sustainable energy planning in Fotokol. On the other hand, the highest and lowest COE values for the PV/FC/Battery hybrid energy system installed in NEOM, Saudi Arabia, were \$0.176/kWh and \$0.126/kWh, respectively [70]. This variation reflects the impact of load profiles and system design parameters on overall system economics.

The high cost of fuel cell systems has been mitigated through innovative approaches, such as utilizing excess energy for hydrogen production, which can be employed in various applications. By harnessing surplus energy from renewable sources like wind or solar, hydrogen can be produced through electrolysis and stored for later use. This hydrogen can then serve as a versatile energy carrier for a variety of applications, including transportation, heating, and power generation, thus enhancing the overall economic feasibility of fuel cell systems. Furthermore, the integration of hybrid storage systems, which combine different forms of energy storage such as batteries and hydrogen storage, can significantly reduce the cost of fuel cell systems. Hybrid storage systems provide greater flexibility and efficiency in managing energy supply and demand. For instance, when renewable energy generation is high, excess electricity can be used to produce hydrogen, while during periods of low energy availability, the stored hydrogen can be used in the fuel cell to provide power. This dynamic approach optimizes the use of available resources, improves system reliability, and reduces the need for expensive standalone storage systems.

### 3.4 Hydrogen fuel production based excess energy

This section will analyze the viability of utilizing surplus energy for hydrogen production. A methodology for utilizing excess energy from renewable energy systems (RESs) aimed at meeting 100% of electrical demand is offered to assess the economic viability of creating hydrogen fuel for local use in industrial and transportation sectors. The hydrogen production per hour $\text{(}M_{{\text{H}}_{2}}(ttext)$ can be determined based on the energy available during that hour:


$$M_{{\text{H}}_{2}}(t)=\frac{P(t)}{\eta \cdot HHV_{{\text{H}}_{2}}}$$

where, $M_{{\text{H}}_{2}}(t)$ is the hydrogen produced during hour t (in kg), P(t) is the power available during hour t  (in kW). $HHV_{{\text{H}}_{2}}$ is the higher heating value of hydrogen (in kWh/kg) and equals 33.6 kWh/kg. moreover, the electrolyzer efficiency is represented by  η and equal 70%.

The total electricity cost for hydrogen production is computed by multiplying the total energy used by the cost of electricity per $kWh\text{(}C_{\text{elec}}\text{)}(\$)$. The total electricity cost is:


$$C_{\text{elec},\text{total}}=E_{\text{total}}\cdot C_{\text{elec}}$$

**Fig 22:**
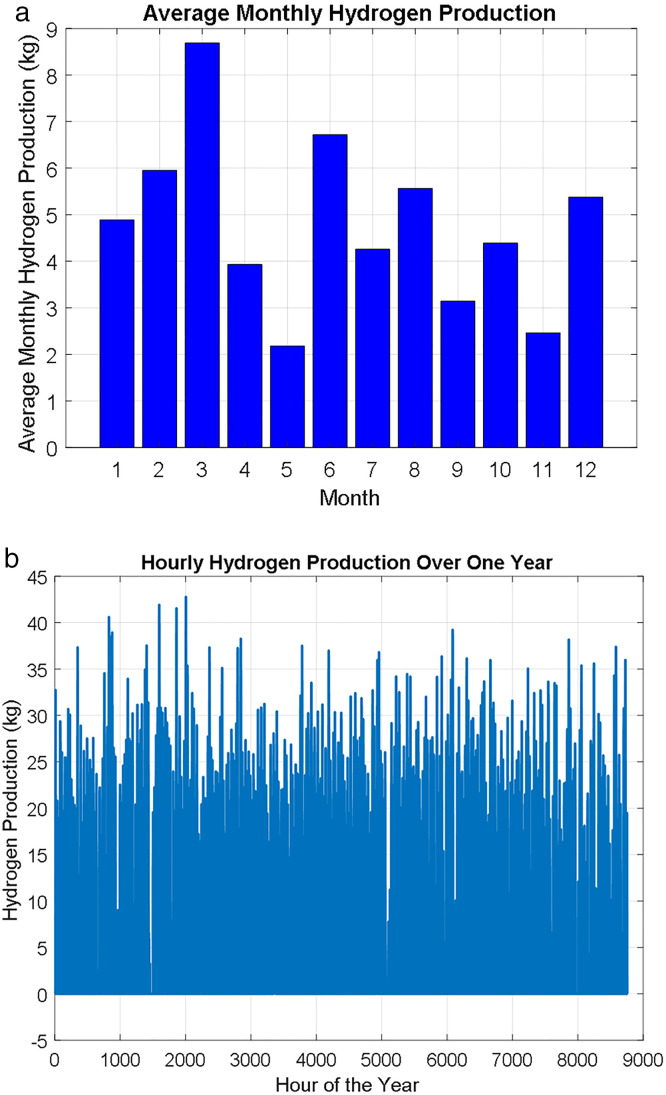
a) Average Monthly Hydrogen Production (kg) and b) Hydrogen Produced over the Year.

The cost per kilogram of hydrogen produced $\text{(}C_{{\text{H}}_{2}}\text{)}$ is then:


$$C_{{\text{H}}_{2}}=\frac{C_{\text{elec},\text{total}}}{M_{{\text{H}}_{2},\text{total}}}$$

where, $C_{\text{elec}}$ is the cost of electricity per kWh (in dollars), $C_{\text{elec},\text{total}}\;$ is the total electricity cost for hydrogen production. The maximum capacity of the hydrogen tank is assumed as 50 kg, if the total hydrogen production exceeds this capacity or in such case of the tank is full, the hydrogen production is capped at 50 kg. Considering the cost of the kWh based the third RES configuration is $0.106/kWh. The results indicate that the cost per kilogram of hydrogen 2.3520$. Moreover, the average hourly hydrogen production is 5.46 kg. the total hydrogen produced over the Year 47800.82 kg. the utilization of the hydrogen in the industrial community, transportation, exportation maximize the value of the 100 green RESs. Fig 22 shows the average monthly hydrogen production (kg) and b) shows the hydrogen production (kg) over the year. The results demonstrate the potential of hydrogen as a flexible energy carrier that enhances system resilience, enables sector coupling, and supports full RES utilization in local industrial and transport applications.

### 3.5 Sensitivity analysis of renewable energy resources and economic parameters

**Fig 23:**
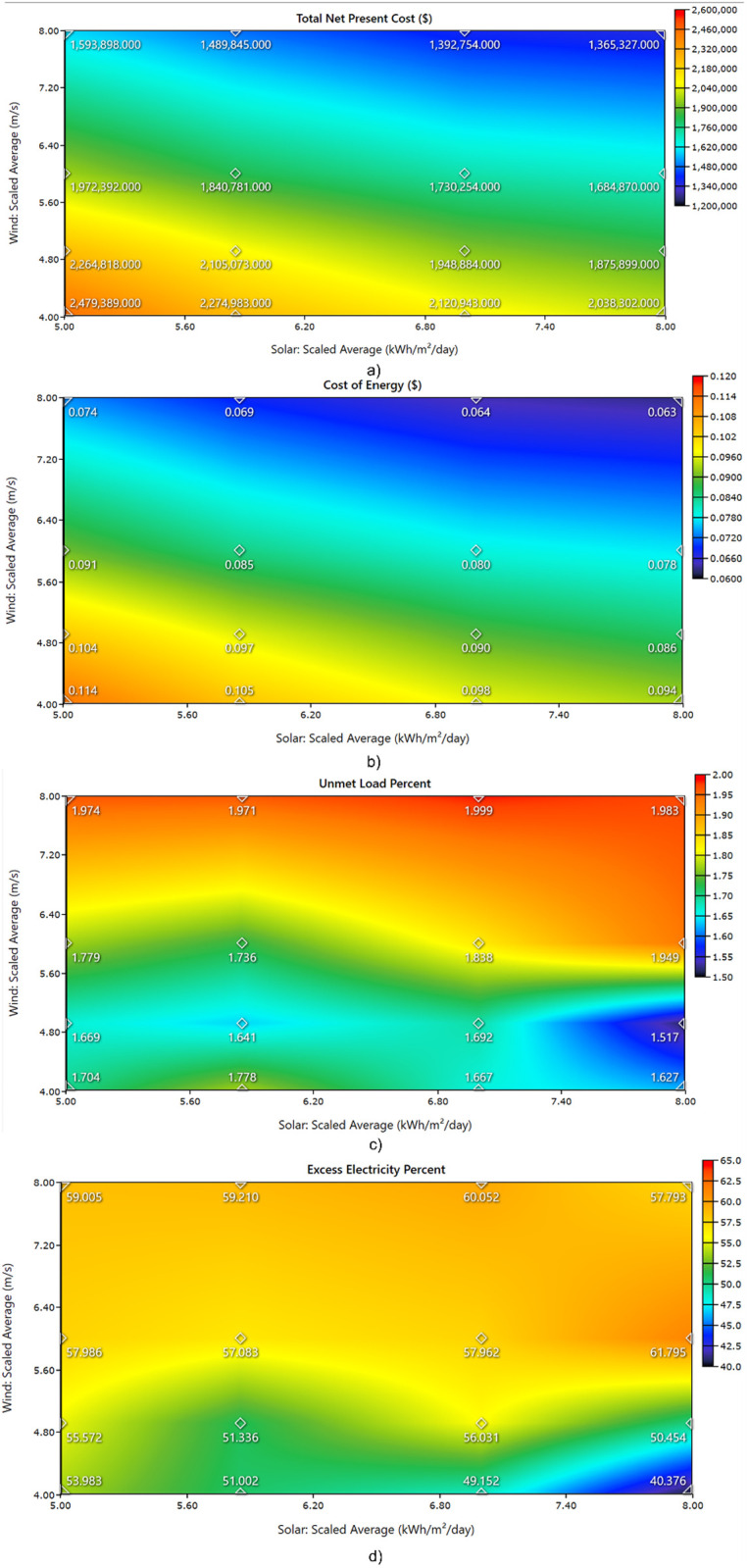
Impact of Solar and Wind Resource Variability on Hybrid Energy System Performance; (a) Total Net Present Cost, (b) Cost of Energy, (c) Unmet Load Percent, and (d) Excess Electricity Percent.

The simulation incorporates various input datasets, including load profiles, component specifications, and meteorological parameters, to evaluate the sensitivity of renewable energy resources. Given the inherent year-to-year variability and uncertainty in these datasets, a range of conditions was analyzed. The sensitivity analysis considering the variation of the average wind speed: 2–10 m/s, and solar radiation: 4–8 kWh/m²/day. Fig 23 illustrates the variation in Net Present Cost (NPC) and Cost of Energy (COE) across these conditions. The Figure shows the variations in solar radiation and wind speed could affect the techno-economic and reliability performance of a hybrid renewable energy system. It includes four performance metrics: total net present cost (NPC), cost of energy (COE), unmet load percent, and excess electricity percent. In Fig 23.a), NPC decreases as both solar and wind resources increase. The lowest NPC occurs when wind speeds exceed 7 m/s and solar radiation exceeds 7 kWh/m²/day, highlighting the economic benefit of high renewable energy potential. Conversely, limited resources in both domains result in a sharp increase in NPC, exceeding $2.4 billion in the worst-case scenario. Moreover, Fig 23.b) shows COE values that range from $0.063 to $0.114. COE drops with higher solar and wind input, particularly when wind exceeds 7 m/s. Systems with low solar and low wind input yield the highest COE values, indicating inefficient energy supply under poor resource conditions. Furthermore, Unmet load percent in Fig 23.c) remains below 2% across most scenarios. High solar input contributes more to reducing unmet load compared to wind. However, a sharp increase appears when wind and solar values are both low, reaching nearly 2%, which indicates challenges in meeting load requirements under poor resource conditions. Fig 23.d) illustrates the excess electricity generated, ranging from 40% to over 60%. Excess energy increases with higher wind speeds and solar radiation, particularly when both exceed mid-range values. Based on the obtained results, one can suggest that without adequate storage or demand-side management, significant portions of generated energy are unused. The results validate the relation between renewable resource availability and system performance. Moreover, the results emphasize the need to balance system sizing, storage integration, and demand response to minimize cost, ensure supply reliability, and reduce energy wastage.

**Fig 24:**
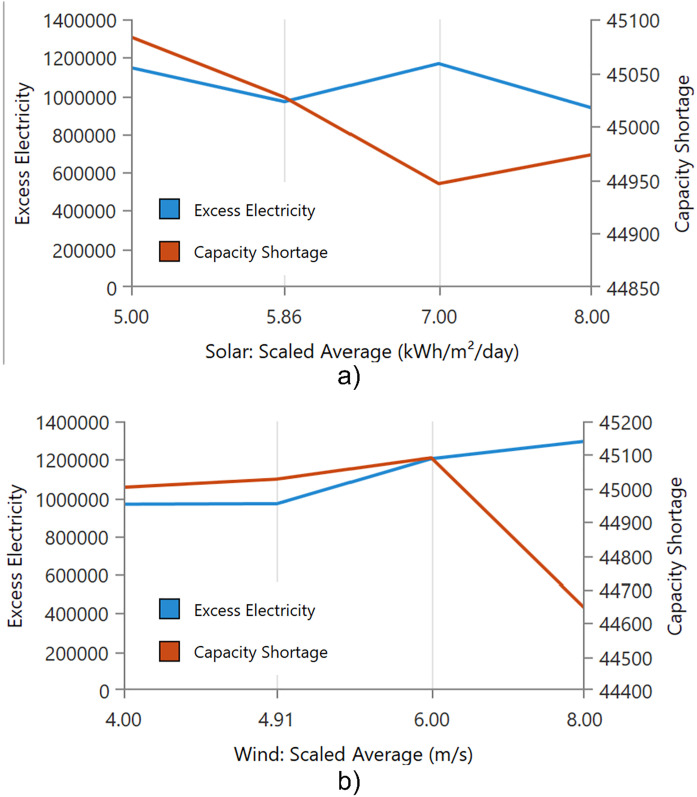
Effect of solar irradiance and wind speed on excess electricity and capacity shortage in a hybrid energy system; a) Variation with average daily solar irradiance (kWh/m²/day) and b) Variation with average wind speed (m/s).

Fig 24 illustrates the impact of varying solar and wind resources on excess electricity and capacity shortage in a hybrid energy system. In Fig 24.a), as the average daily solar irradiance increases from 5.0 to 8.0 kWh/m²/day, excess electricity exhibits a non-linear trend. It initially decreases, peaks at approximately 7.0 kWh/m²/day, and then slightly drops at the highest irradiance level. In contrast, capacity shortage decreases sharply with increasing solar availability until 7.0 kWh/m²/day, after which it slightly increases. This suggests that higher solar availability enhances system reliability up to an optimal point, beyond which the marginal gains in reliability decline and excess energy generation may be curtailed due to system limitations. In Fig 24.b), the effect of wind speed variation on the same two metrics is shown. As wind speed increases from 4.0 to 8.0 m/s, excess electricity steadily increases, indicating higher generation potential. Capacity shortage remains relatively stable between 4.0 and 6.0 m/s but then drops significantly at 8.0 m/s. This sharp decline indicates that wind energy becomes more effective at meeting demand and reducing supply gaps under high-resource conditions. Compared to solar, wind energy demonstrates a more consistent positive effect on both excess electricity and system reliability. The results focus the reputation of resource quality in the performance of hybrid systems. Solar energy contributes to reliability up to a certain irradiance threshold, while wind energy shows a clearer and stronger benefit at higher speeds. Moreover, there is need for balanced resource integration and careful dispatch strategy to minimize curtailment and ensure stable operation.

**Fig 25:**
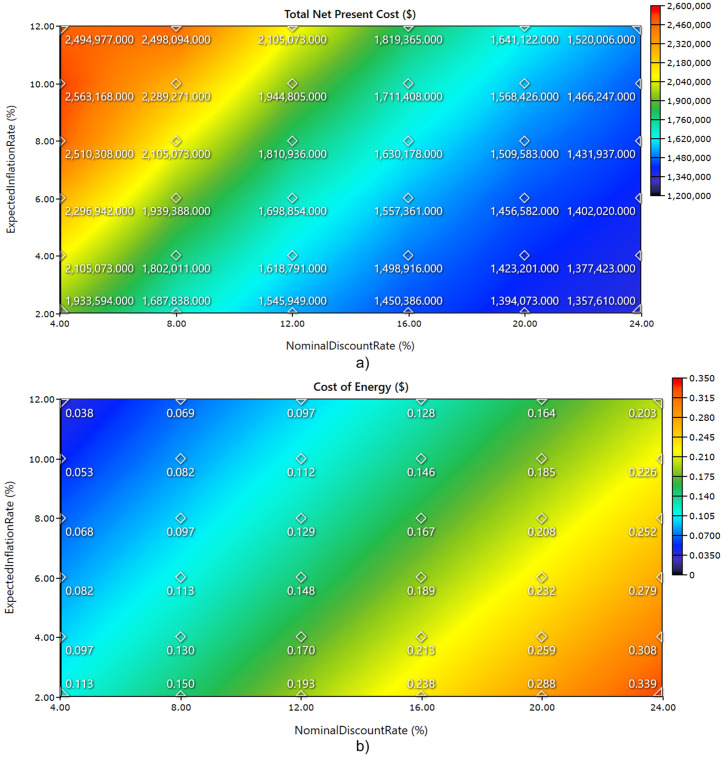
Impact of Nominal Discount Rate and Expected Inflation Rate on Hybrid Energy System Performance, (a) Total Net Present Cost, (b) Cost of Energy.

**Fig 26:**
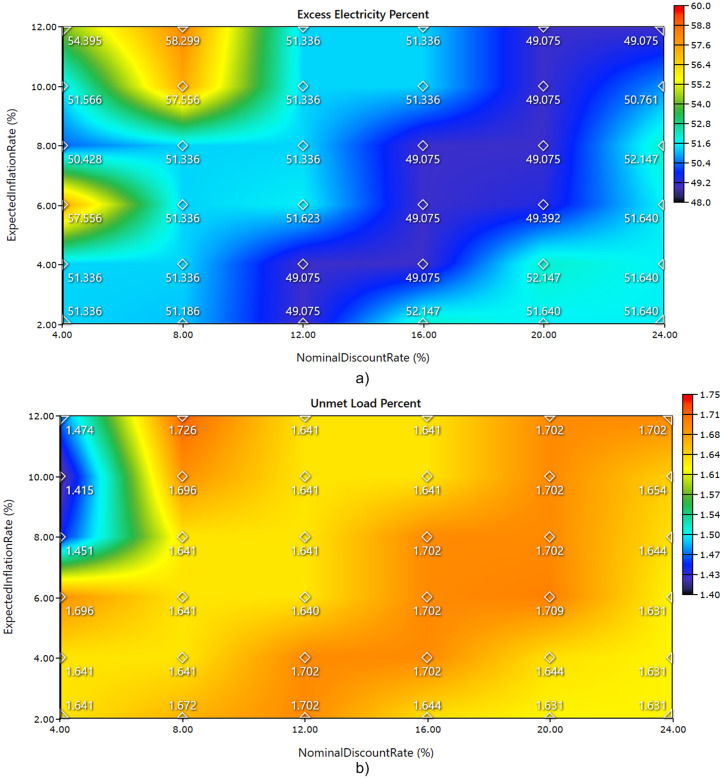
Impact of nominal discount rate and expected inflation rate on the reliability performance of the hybrid energy system performance (a) Excess Electricity Percent, and (b) Unmet Load Percent.

The economic uncertainty analysis has been conducted. Estimating future financial values in developing countries is complicated by volatility in discount rates and inflation levels. To capture the range of likely economic scenarios, both lower and upper bounds of these parameters were included in the analysis as the discount rate: 4–24% and the inflation rate: 2–12%. Figs 25 and 26 illustrate illustrates the influence of nominal discount rate and expected inflation rate on the techno-economic and reliability performance of a hybrid energy system through four key indicators: total net present cost (TNPC), cost of energy (COE), excess electricity percent, and unmet load percent. In Fig 25.a), TNPC decreases as both discount rate and inflation rate increase. The lowest TNPC occurs at the highest discount (24%) and inflation (12%) combination, while the highest TNPC is observed under low discount and inflation rates, indicating the sensitivity of long-term investment costs to financial assumptions. Fig 25.b) shows a similar trend for COE, with lower values occurring at lower financial rates and higher COE values when both discount and inflation rates rise, peaking above $0.33/kWh. This increase in COE is driven by the cost amplification effect of financial parameters on the capital-intensive renewable energy system. Fig 26.a) reveals that excess electricity remains relatively stable across most of the financial scenarios, clustering around 49–52%, with notable peaks exceeding 57% at low discount and high inflation settings. This may reflect system oversizing or lower utilization under those financial conditions. Fig 26.b) demonstrates that unmet load percent varies only slightly across the entire matrix, remaining close to 1.64%, except for minor deviations under extreme financial assumptions. The results confirm that the system maintains a high level of reliability regardless of changes in economic parameters, while economic performance metrics show greater sensitivity. Moreover, the importance of incorporating realistic financial variables into the planning process of renewable-based hybrid systems has been validated.

### 3.6 Policy, integration, and practical relevance

The analyzed configurations must not only meet technical and economic benchmarks but also align with national energy strategies and grid integration priorities. Saudi Arabia’s Vision 2030 emphasizes renewable energy expansion, hydrogen economy development, and reduced dependence on fossil fuels. The proposed configurations, particularly Configuration 3 with hydrogen integration, directly support these long-term policy goals.

Configuration 3 demonstrates strategic relevance by converting excess renewable energy into hydrogen via electrolysis, aligning with the National Hydrogen Strategy and Saudi Arabia’s ambition to become a leading hydrogen exporter. However, realizing such systems at scale requires alignment with existing investment frameworks, infrastructure availability, and regulatory readiness for hydrogen handling, transport, and storage.

From a grid perspective, the high levels of excess electricity in all configurations highlight the importance of incorporating smart grid solutions, demand-side management, or grid-export capabilities. The absence of grid-tied operation in the current model simplifies analysis but limits its practical applicability, especially in regions with available grid access or future expansion plans.

Operationally, harsh desert conditions typical of Saudi Arabia pose additional challenges. Components like batteries, wind turbines, and electrolyzers must be evaluated for thermal performance, dust tolerance, and maintenance logistics. These real-world constraints affect system reliability, lifecycle cost, and deployment feasibility.

Economically, although Configuration 2 shows the lowest LCOE, Configuration 3 introduces energy vector diversification, which supports sector coupling—enabling hydrogen use in transport, industry, or seasonal storage. This increases long-term value, especially under future carbon pricing or fuel export scenarios.

- For deployment readiness, future models should incorporate:
- Grid interconnection policies and tariffs
- Hydrogen regulatory codes and safety standards
- Market-based incentives or subsidies
- Realistic component degradation and replacement cycles
- Geographic and socio-economic factors of the selected region

Integrating these practical dimensions will provide deeper insight into the real-world feasibility and strategic value of hybrid renewable energy systems in line with national goals.

## 4. Conclusions

This study conducted a comparative analysis of three renewable energy systems: PV/battery, PV/wind turbine (WT)/battery storage system (BSS), and PV/WT/BSS/electrolyzer/fuel cell (FC), specifically tailored for commercial applications in Neom, Saudi Arabia. The analysis conducted using HOMER software identified the optimal configurations, component sizes, and associated costs, emphasizing the minimization of the cost of energy (COE) and net present cost (NPC). The findings demonstrate that substituting the battery bank with an electrolyzer, fuel cell, and hydrogen tank increases the net present cost (NPC) owing to the elevated expenses associated with these components. However, the PV/WT/BSS/electrolyzer/FC configuration resulted in the lowest unmet electric load of 16,724 kWh/year and highest total energy production of 1,475,041 kWh/year, yielding 547,960 kWh/year of surplus electricity. This configuration resulted in the highest NPC of $1.52 million and a COE of $0.137 per kWh. The PV/BSS and PV/WT/BSS configurations demonstrated lower net present costs (NPCs) of $1.17 million and $1.27 million, respectively, with corresponding costs of energy (COEs) of $0.116 per kilowatt-hour and $0.106 per kilowatt-hour. The PV/BSS and PV/WT/BSS systems produced 1,344,029 and 1,273,402 kWh/yr, respectively, generating excess electricity values of 431,362 and 374,345 kWh/yr, respectively. The PV/WT/BSS system demonstrated optimality for applications that prioritize cost efficiency and minimal operational expenses because of its lower cost. The PV/WT/BSS/Elec/FC system, although more expensive, is more appropriate for projects requiring greater energy storage capacity and reliability, particularly in off-grid or energy-independent contexts. The findings indicate that the PV/WT/BSS configuration is the most cost-effective solution, whereas the PV/WT/BSS/Elec/FC system is a viable alternative for projects requiring enhanced energy reliability. Future work will focus on implementing real-time dispatch algorithms that co-optimize renewable availability, electrolyzer dynamics, fuel cell performance, and storage state-of-charge, while incorporating hydrogen leakage, pressure dynamics, and system degradation to enhance operational realism and model validity. Moreover, the future research should also focus on optimizing hydrogen storage and fuel cell technologies, particularly in relation to electric vehicles (EVs) and hydrogen-powered vehicles, to lower costs and improve the economic viability and performance of PV/WT/BSS/Elec/FC systems. The application of advanced optimization techniques to further validate the optimality of the proposed hybrid energy system. These techniques will be compared with HOMER’s optimization results to assess their impact on system performance, cost-efficiency, and scalability in different configurations.

## Supporting information

S1 Table Dataset of results for the first configuration. (CSV)

S2 Table Dataset of results for the second configuration. (CSV)

S3 Table Dataset of results for the third configuration. (CSV)

## Abbreviations

GHG: Greenhouse Gas
ESS: Energy Storage System(s)
HES: Hybrid Energy System(s)
PV: Photovoltaic
BSS: Battery Storage System
WT: Wind Turbine
FC: Fuel Cell
NPC: Net Present Cost
COE: Cost of Energy
LCOE: Levelized Cost of Energy
LPSP: Loss of Power Supply Probability
KSA: Kingdom of Saudi Arabia
RES: Renewable Energy Source(s)
SG: Smart Grid
HOMER: Hybrid Optimization of Multiple Energy Resources
HRES: Hybrid Renewable Energy System(s)
PEMFC: Proton Exchange Membrane Fuel Cell
PSO: Particle Swarm Optimization
DG: Diesel Generator
LCOH: Levelized Cost of Hydrogen
COH: Cost of Hydrogen
CSA: Cuckoo Search Algorithm
O&M: Operation and Maintenance
MG: Microgrid
STC: Standard Test Conditions
NOCT: Nominal Operating Cell Temperature
SoC: State of Charge
DC: Direct Current
AC: Alternating Current
$P_{PV}$: PV Power Output
$Y_{PV}$: Rated Capacity of the PV Array
$f_{PV}$: PV Derating Factor
$G_{T}$: Global Solar Radiation Incident on the PV Array
$G_{T,STC}$: Standard Incident Radiation under STC
$\alpha_{P}$: Temperature Coefficient of Power
$T_{C}$: PV Cell Temperature under Operating Conditions
$T_{C,STC}$: PV Cell Temperature under STC
$T_{a}$: Ambient Temperature
V: Wind Speed
$V_{1}$: Wind Speed at Reference Height h₁
$V_{2}$: Wind Speed at Hub Height h₂
$h_{1}$: Reference Height
$h_{2}$: Hub Height
γ: Friction Coefficient
$P_{WT}$: WT Power Output
$P_{r}$: Rated Power
$V_{cut-in}$: Cut-in Wind Speed
$V_{rated}$: Rated Wind Speed
$V_{cut-out}$: Cut-out Wind Speed
$C_{p}$: Maximum Power Coefficient
$\rho_{air}$: Air Density
$A_{wind}$: Blade Swept Area
$\sigma_{b}$: BSS Self-Discharge Rate
$\eta_{inv}$: Inverter Efficiency
$\eta_{C}$: Charging Efficiency of the BSS
$\eta_{BD}$: Discharging Efficiency of the BSS
$P_{L}$: Electrical Load Demand
Δt: Time Step
$SoC_{\max}$: Maximum State of Charge
$SoC_{\min}$: Minimum State of Charge
$H_{2}$: Hydrogen Gas
$O_{2}$: Oxygen Gas
$OH^{-}$: Hydroxide Ion
$e^{-}\;$: Electron
$H^{+}$: Hydrogen Ion/ Proton
$H_{2}O$: Water
$P_{EL}$: Electrolyzer Power Consumption
$\dot{m_{H_{2}}}$: Hydrogen Flow Rate
$HHV_{H_{2}}$: Higher Heating Value of Hydrogen
$\eta_{EL}$: Electrolyzer Efficiency
$V_{FC}$: Fuel Cell Voltage Output
$E_{rev}$: Reversible Voltage
$V_{act}$: Activation Voltage Loss
$V_{diff}$: Diffusion Voltage Loss
$V_{\Omega }$: Ohmic Voltage Loss
$P_{inv}$: Converter/ Inverter Capacity
$EL_{\max}$: Maximum Electrical Load Demand
$\eta_{dc\text{/}ac}$: Efficiency for DC-to-AC Conversion
$\eta_{ac\text{/}dc}$: Efficiency for AC-to-DC Conversion
$P_{BSS,ch\text{/}dis}$: Charge/Discharge Power of the BSS
$P_{G}$: Imported/Exported Grid Power.
